# A *Burkholderia* phage selects for attenuated virulence and antimicrobial hypersensitivity through increased outer membrane permeability

**DOI:** 10.3389/fmicb.2026.1876186

**Published:** 2026-07-09

**Authors:** Philip Lauman, Nora A. S. Hussain, James L. Stafford, Jonathan J. Dennis

**Affiliations:** 1 Faculty of Land and Food Systems, University of British Columbia, Vancouver, BC, Canada; 2 Department of Biological Sciences, Faculty of Science, University of Alberta, Edmonton, AB, Canada

**Keywords:** bacteriophage, *Burkholderia gladioli*, lipopolysaccharide, membrane permeability, phage immunogenicity, phage steering, phage therapy, phage-antibiotic synergy

## Abstract

*Burkholderia gladioli* is an opportunistic pathogen with intrinsic antimicrobial resistance and is therefore a compelling target for phage therapy (PT), yet bacteriophages infecting this species remain largely uncharacterized. Here, we show that the functionally lytic (FL), lipopolysaccharide (LPS)-binding myovirus KS12, originally isolated against *Burkholderia cenocepacia*, suppresses *B. gladioli* growth *in vitro* and infection-associated mortality in *Galleria mellonella*. KS12 selects for resistant subpopulations carrying mutations in the *O*-antigen biosynthesis and export pathways that compromise outer membrane (OM) integrity, resulting in attenuated virulence *in vivo* and hypersensitivity to human serum, antimicrobial peptides, and polymyxins. Consistent with this trade-off, KS12 and colistin interact synergistically to substantially reduce bacterial densities, suggesting that predation by KS12 may impose an evolutionary trap on *B. gladioli*. Furthermore, comparative analyses indicate that outer membrane permeability correlates strongly with colistin susceptibility across Gram-negative pathogens, implying that antivirulence steering with LPS-binding phages could provide a strategy to sensitize intrinsically resistant pathogens to antibiotics of last resort. Although KS12 particles were inactivated by innate humoral immunity, they did not appear to be degraded by or stimulate pro-inflammatory responses in phagocytes, indicating that the antibacterial activity of this phage is not driven by direct immunostimulation. Together, these results identify KS12 as a promising phage targeting *B. gladioli* and highlight the potential of LPS-binding phages to steer bacterial populations toward attenuated virulence and hypersensitivity, thereby yielding more clinically tractable phenotypes.

## Introduction

The widespread overuse of antibiotics has driven the emergence of resistance, leading to eroded antimicrobial efficacy, a rise in untreatable infections, and increased interest in alternative antibacterial strategies (O’Neill, 2014; Clancy and Hong Nguyen, 2020; Naghavi et al., 2024). Key among these approaches is phage therapy (PT), which utilizes bactericidal viruses (bacteriophages) to infect and eliminate bacterial pathogens selectively (Abedon et al., 2011; Schooley et al., 2017; Pirnay et al., 2024). In particular, recent work has focused on antivirulence phage steering, a strategy wherein phage predation is leveraged as a selective pressure to bias bacterial populations toward more clinically tractable phenotypes (Gordillo Altamirano et al., 2021; Lauman and Dennis, 2021). In parallel, increasing attention has been directed toward the immunological consequences of phage exposure and whether phages stimulate host responses that influence therapeutic outcomes (Krut and Bekeredjian-Ding, 2018; Le et al., 2025).

Bacteriophages in the order *Caudoviricetes* are invariably capable of replication through the lytic cycle, in which injected viral genomes are expressed to produce progeny virions that subsequently lyse the bacterial cell, and phages that can replicate *only* in this manner are dubbed obligately lytic (OL; Salmond and Fineran, 2015; Hobbs and Abedon, 2016). In contrast, temperate or lysogeny-capable (LC) phages can alternatively establish lysogens by integrating their genomes into host chromosomes or persisting as plasmid-like elements, allowing passive replication through vertical transmission. Although LC phages may be suitable in certain contexts (Howard-Varona et al., 2017; Al-Anany et al., 2021; Lauman and Dennis, 2023; Cook and Hynes, 2025), OL phages are generally considered preferable for therapeutic applications due to their superior safety and bactericidal activity (Skurnik and Strauch, 2006; Fortier and Sekulovic, 2013). Genomic analyses are typically used to confirm OL replication, but sequencing is sometimes impracticable for certain phages with extensively modified DNA. In such cases, phages that consistently exhibit lytic behavior and fail to establish resistance via lysogeny can be described as functionally lytic (FL), reflecting effective bactericidal activity despite incomplete genomic characterization (Kieft et al., 2020; Cook and Hynes, 2025; Chopjitt et al., 2026).

Predation by lytic phages imposes strong fitness costs on susceptible bacteria, and these viruses therefore often select for resistant subpopulations with modified or absent receptor structures (Oechslin, 2018; Lauman and Dennis, 2021). Since many phages utilize lipopolysaccharide (LPS), capsule components, pili, flagella, adhesins, efflux pumps, and other virulence or antimicrobial resistance determinants as receptors, phage predation tends to steer bacterial populations toward antibiotic-sensitized and virulence-attenuated phenotypes (Chan et al., 2016; McCutcheon et al., 2018; Gordillo Altamirano et al., 2021; Gao et al., 2022; Menon et al., 2022; Zulk et al., 2022; Ruest et al., 2023; Supina et al., 2025).

*Caudoviricetes* virions are functionally and morphologically distinct from those of eukaryotic viruses, yet many structural features of their capsids and genomes are conserved and can be recognized by host immune sensors (Yutin et al., 2018; Koonin et al., 2020). Indeed, recognition of phage capsid proteins and dsDNA has been shown to trigger diverse immune responses in multiple mammalian cell types (Shepardson et al., 2017; Gogokhia et al., 2019; Podlacha et al., 2024; Le et al., 2025). Nevertheless, the immunological consequences of phage exposure are not yet fully characterized, with divergent findings reported across experimental systems (Miernikiewicz et al., 2013; Mirzaei et al., 2016; Shiley et al., 2017; Van Belleghem et al., 2017; Freyberger et al., 2018; Cafora et al., 2021; Le et al., 2025), and their relevance to PT remains a subject of considerable debate. In parallel, although antibody-mediated neutralization of certain phages has been described (Zaczek et al., 2016; Krut and Bekeredjian-Ding, 2018; Rouse et al., 2020), the roles of innate humoral defenses and professional phagocytes in phage inactivation remain incompletely understood.

Interestingly, *in vivo* phage titer decline in the absence of adaptive immunity has been observed to coincide with effective bacterial clearance (Seed and Dennis, 2009; Semler et al., 2014; Lauman and Dennis, 2021). Given that stimulation of innate pro-inflammatory responses can promote non-specific phagocytosis and degradation of both bacteria and phages (Akira et al., 2006), we speculated that phage-mediated immunostimulation might simultaneously enhance the innate immune system’s clearance of phages and bacteria.

*Burkholderia gladioli*, a relative of the notorious *Burkholderia cepacia* complex (Bcc), is an emerging opportunistic pathogen that can cause severe pneumonia and septicemia in individuals with cystic fibrosis (CF), chronic granulomatous disease (CGD), and other immunocompromising conditions (Boyanton et al., 2005; LiPuma, 2010; Quon et al., 2011; Thompson et al., 2011; Brizendine et al., 2012; Gauvreau et al., 2023). *B. gladioli* exhibits high intrinsic resistance to many antimicrobials, and infection with this pathogen is often considered a contraindication for lung transplantation, leaving affected patients with few therapeutic options (Murray et al., 2008; Segonds et al., 2009; Dursun et al., 2012; Somayaji et al., 2020). Moreover, *B. gladioli* causes a broad range of diseases in both crops and wild plants, and environmental reservoirs of this pathogen have been implicated in several cases of human acquisition and infection (Compant et al., 2008; LiPuma, 2010; Jones et al., 2021).

Although *B. gladioli* represents a compelling target for the development of PT, very few phages infecting this species have been characterized (Duplessis et al., 2019; Wang et al., 2024; Britton and Dennis, 2026), and to our knowledge, no studies have systematically examined the antibacterial efficacy, antivirulence-steering potential, or immunogenicity of a phage capable of lysing *B. gladioli*. Here, we show that KS12, an FL *Burkholderia* myovirus, suppresses *B. gladioli* growth *in vitro* and infection-associated mortality *in vivo*. As an LPS-binding phage, KS12 selects for survivor populations with compromised outer membranes (OMs), which consequently exhibit attenuated virulence and hypersensitivity to serum and cationic antimicrobials; therefore, it exhibits potent antibacterial synergy with colistin. Although KS12 is readily inactivated by humoral innate immunity, it does not appear to independently stimulate pro-inflammatory immune responses and interacts minimally with professional phagocytes. Together, our findings show that KS12 has potential as a therapeutic agent targeting *B. gladioli* and demonstrate that LPS-binding phages can steer bacterial populations toward susceptibility to immune effectors and antibiotics of last resort.

## Materials and methods

### Organisms and media

Twelve strains of *B. gladioli* were examined, including eight clinical isolates from Canadian (*n* = 7) and British (*n* = 1) CF patients, one isolate from an American CGD patient, and three isolates from rhizosphere samples. Additionally, *B. cenocepacia* clinical isolates C6433, Van1, K56-2, and two LPS mutants of the latter strain, along with *Stenotrophomonas maltophilia* SMDP92, *Pseudomonas aeruginosa* PA01, and *Acinetobacter baumannii* AB5075, were used for specific analyses (Supplementary Table S1). Bacteria were incubated statically on LB Lennox 1.5% w/v agar at 30 °C for 48 h, and single colonies were cultured in 5 mL LB Lennox broth at 30 °C, shaking at 225 rpm, for 18 h. Cultures were then plated for viable colony-forming units (CFUs), and optical density at 600 nm (OD_600_) was measured using a NanoDrop 2000c spectrophotometer (ThermoFisher Scientific, Waltham, MA, USA). Strain-specific OD_600_-CFU standard curves were generated and used to estimate viable CFUs in 18 h cultures, which were diluted in LB Lennox as required. For long-term storage, strains were maintained at −80 °C in LB Lennox supplemented with 20% glycerol.

RAW 264.7 murine macrophages (ATCC TIB-7) were cultured in Dulbecco’s Modified Eagle Medium (DMEM; Cytiva, Marlborough, MA, USA) supplemented with 5% Fetal Bovine Serum (FBS; FisherBrand, Pittsburgh, PA, USA) and 100 U penicillin + 100 µg mL^−1^ streptomycin (Gibco, Carlsbad, CA, USA) as previously described (Phillips et al., 2020). Cells were incubated at 37 °C, 5% CO_2_, and passage into 75 cm^2^ vented flasks (Corning, Corning, NY, USA) every other day by scraping off and subculturing cells roughly 1:10 in fresh medium.

Ten *Burkholderia* phages, previously isolated and partially characterized in the Dennis Lab, were used in this study (Supplementary Table S2), including eight experimentally LC phages (AH2, DC1, JC1, KL1, KS4M, KS5, KS9, and KS14), and OL and FL phages JG068 and KS12, respectively. Phages were stored at 4 °C in suspension medium (SM; 50 mM Tris–HCl [pH 7.5], 100 mM NaCl, 8 mM MgSO_4_), and titers were routinely verified. Phages were amplified using the double-layer agar approach (Kamal and Dennis, 2015), lysate containing phages was resuspended in 3 mL of SM, scraped off plates, and centrifuged at 12,000 *g* for 7 min, and the supernatant was sterilized using a 0.45 μ m Mixed Cellulose Ester (MCE) filter (Merck Millipore, Ireland) and titers were verified using the double-layer agar method. Phage amplifications were performed using *B. cenocepacia* C6433 (for AH2, DC1, KL1, KS4M, KS5, and KS14), K56-2 (JG068, KS9, and KS12), and Van1 (JC1), and were repeated to maintain phage titers ≥ 10^9^ plaque-forming units (PFUs) mL^−1^.

*Galleria mellonella* larvae were reared in-house as described previously (Seed and Dennis, 2008, 2009), but with substantial modifications. Briefly, 20 larvae were placed into mesh-sealed mason jars containing 250 g of nutrient mix (282 g kg^−1^ wheat germ, 141 g kg^−1^ brewer’s yeast, 224 g kg^−1^ beeswax, 141 g kg^−1^ glycerol, 141 g kg^−1^ honey, 71 mL kg^−1^ MilliQ H_2_O). They were incubated at 30 °C in the dark until larvae converted into moths, laid eggs, and the eggs hatched into larvae. Hatched larvae were placed into plastic containers with 1 kg nutrient mix and were stored at room temperature (RT) until maturation (Tsai et al., 2016). Mature larvae ( ≈ 0.3 g; 2.5 cm in length) were used immediately, and unused larvae were euthanized via freezing (−20 °C overnight) followed by autoclaving at 121 °C for 30 min.

Aside from chloramphenicol and ciprofloxacin, which were, respectively, dissolved in 100% ethanol and 0.1 M HCl, all antibiotics, antimicrobial peptides, and surfactants were prepared in MilliQ H_2_O at a concentration of 10 mg mL^−1^ (Supplementary Table S3). Stocks were sterilized using 0.45 μm MCE filters (Merck Millipore, Ireland) and diluted to the required concentrations in LB Lennox.

### Host range and infectivity assays

Host range and efficiency of phage activity (EPA) assays were conducted as described previously (Lauman and Dennis, 2023), with minor modifications. Briefly, lawns of bacterial target strains were poured onto LB Lennox 1.5% w/v agar plates using the double-layer agar method, and 5 μ L of phage (ranging from 10^10^ to 10^3^ PFU mL^−1^) was spotted onto lawns. Plates were inspected for evidence of infection (plaques, mottling, or turbidity) after 18 h of incubation at 30 °C, and strains that showed no evidence of infection at *any* concentration were deemed insensitive. For sensitive strains, EPA was calculated as defined previously for each phage-host pair (Lauman and Dennis, 2023), and pairs exhibiting EPA ≥ –2 were investigated further.

### Planktonic killing assays

Planktonic killing (PK) assays were conducted and analyzed as described previously (Lauman and Dennis, 2023; Lauman et al., 2026), with minor modifications. Briefly, 0.1 mL of bacterial culture, adjusted to 10^6^ CFU mL^−1^ in LB Lennox or glucose-supplemented M9 minimal medium (MM; 6 g/L Na_2_HPO_4_, 3 g/L KH_2_PO_4_, 0.5 g/L NaCl, 1 g/L NH_4_Cl, 1 mM MgSO_4_, 0.1 mM CaCl_2_, 2% w/v glucose), was combined with 0.1 mL of phage, diluted in SM to concentrations between 10^5^ and 10^9^ PFU mL^−1^, in a flat-bottomed 48-well plate (Corning, Corning, NY, USA), yielding starting multiplicities of infections (MOIs) ranging from 0.1 to 1,000 PFU CFU^−1^. Following a 10 min incubation at RT, 0.8 mL of LB Lennox or M9 MM was added to each well. Plates were incubated in an Epoch 2 microplate spectrophotometer (BioTek, Winooski, VT, USA) at 30 °C with continuous shaking (237 rpm), and OD_600_ readings were recorded automatically hourly for 48 h. Definite integrals of OD_600_-time curves were computed using the trapezoid approximation method, and growth reduction coefficient (GRC) values were computed as previously defined for each phage-host pair across all tested MOIs (Lauman and Dennis, 2023). Phage-host pairs exhibiting GRCs ≥ 0.75 at one or more tested MOIs were investigated further.

### *Galleria mellonella* infection and rescue assays

Infection and phage rescue assays with *G. mellonella* larvae were conducted as described previously (Seed and Dennis, 2008, 2009; Kamal and Dennis, 2015), with minor modifications. Larvae were anesthetized by icing and were injected in the hindmost left proleg, using a PB600-1 dispenser fitted with a 22-gauge needle (Hamilton, Reno, NV, USA), with 5 μ L of 1 × Phosphate-Buffered Saline (PBS) or bacterial cultures adjusted in PBS to 2 × 10^5^ CFU mL^−1^, yielding an inoculum dose of 1 × 10^5^ CFU. Larvae were incubated at 30 °C for 2 h, anesthetized, and injected with 10 μ L of SM or phages diluted in SM to 1 × 10^9^ PFU mL^−1^, yielding a treatment dose of 1 × 10^7^ PFU (MOI 100). A 10x lower inoculum was used to achieve greater temporal resolution in studies comparing the relative virulence of phage-resistant mutants, in which no treatment injection was given. Larvae were incubated at 30 °C for up to 120 h post-infection (hpi), and were considered dead if they produced no autonomous responses to physical stimuli when monitored every 24 hpi.

### Quantitative analysis of *in vivo* mortality

In *G. mellonella* infection and rescue assays, percent mortality was defined as the percentage of larvae in a replicate group (n = 10) that were dead and was computed for each timepoint. Maximal time of death ( $t_{D}$ ) was defined as the earliest timepoint with a mean percent mortality ≥ 85% in at least one experimental group, and was considered the upper boundary for computing the definite integral ( A ) of the experimental percent mortality-time curve:


$$A=\int_{t_{0}}^{t_{D}}(\%\text{mortality})\;\mathrm{dt}$$

The maximum value of A ( $A_{\max}$ ) was calculated using a theoretical curve, which has maximum percent mortality at all non-zero timepoints, and the mortality coefficient ( $C_{m}$ ) was defined as the unitless quotient of these integrals:


$$C_{m}=\frac{A}{A_{\max}}$$

The reduction in the mortality coefficient of treated animals ( $C_{m}T$ ) relative to that of untreated animals ( $C_{m}\mathrm{UT}$ ) was quantified by the reduction of the mortality coefficient ( $R\;C_{m}$ ), a unitless value defined as the difference between these coefficients normalized to the mortality coefficient of untreated animals:


$$R\;C_{m}=\frac{C_{m}\mathrm{UT}-C_{m}T}{C_{m}\mathrm{UT}}$$

### Resistant mutant isolation, genome extraction, and sequencing

KS12-resistant (KS12^**R**^) colonies of *B. gladioli* R1879 were isolated from additional replicates of PK assays. Briefly, 1 mL of cell suspension was collected after 48 h of shaking (237 rpm) co-incubation with KS12 (MOI 100) at 30 °C, and was centrifuged and washed (12,000 *g*, 5 min, 0.5 mL LB Lennox) three times to remove phage particles. Cells were resuspended in 1 mL LB Lennox and streaked onto LB Lennox 1.5% w/v agar plates for single colony isolation. Resistance to KS12 was confirmed through comparative double-layer agar and spotting assays on lawns of R1879 wild-type (wt) and three separate survivor isolates: R1879- φ KS12-R1, R1879- φ KS12-R2, and R1879- φ KS12-R3.

Genomic DNA (gDNA) was extracted from purified colonies of R1879 wt and all three survivor isolates using the cetyltrimethylammonium bromide (CTAB) method described previously (Moore et al., 2004), with minor modifications. Briefly, colonies were resuspended in TE buffer containing 0.5% sodium dodecyl sulfate (SDS) and 1 mg mL^−1^ proteinase K, incubated at 37 °C for 1 h, combined with 0.1 mL 5 M NaCl and 0.1 mL heated CTAB solution (10% CTAB in 0.7 M NaCl), and incubated at 55 °C for 10 min. gDNA was subsequently purified using the phenol-chloroform method followed by ethanol precipitation, as described previously (McCutcheon et al., 2020), and was resuspended in sterile MilliQ H_2_O. gDNA quality ( ≥ 120 ng μ L^−1^; A260/A280 ≈ 1.8; A260/A230 ≈ 2.1) was confirmed using a NanoDrop 2000c spectrophotometer (ThermoFisher Scientific, Waltham, MA, USA). Libraries were prepared using the Illumina DNA Prep kit, and high-throughput sequencing was performed using a 300-cycle configuration (2 × 150 bp paired-end reads) on the Illumina NextSeq 2000 platform (Illumina, San Diego, CA, USA) by SeqCenter (Pittsburgh, PA, USA).

### Identification of resistance determinants

Raw reads were quality checked using FastQC v0.12.1 (Babraham Bioinformatics, 2010), and adapters and low-quality bases were trimmed using Trim Galore! v0.6.5 (Babraham Bioinformatics, 2012). Bacterial genomes were *de novo* assembled using Unicycler v0.5.0 (Wick et al., 2017), functioning as an optimizer for SPAdes v4.2.0 (Bankevich et al., 2012), and open reading frames (ORFs) were annotated using Prokka v1.15.5 (Seemann, 2014). SNPs and indels were identified with Snippy v4.6.0 (Seemann, 2015), using the R1879 wt genome as a reference, and the Basic Local Alignment Search Tool (BLASTp) was used to identify proteins affected by coding sequence (CDS) variations (Camacho et al., 2009). Cell-surface-expressed structures encoded by genes or loci affected in all three phage-resistant isolates were inferred to be the determinants of resistance. Genome assembly quality was confirmed using the Comprehensive Genome Analysis Service on the Bacterial and Viral Bioinformatics Resource Center (BV-BRC) server (Olson et al., 2023). SnapGene (Insightful Science, San Diego, CA, USA) was used for genome visualization and construction of genomic maps. All software was run under default parameters.

### Outer membrane permeability assays

Outer membrane permeability assays were performed using nitrocefin as described previously (Balder et al., 2013; Yu and Zhao, 2020), with minor modifications. Briefly, 1 mL of bacterial suspension, subcultured to 5.0 × 10^8^ CFU mL^−1^, was centrifuged and washed (5,000 *g*, 2 min, 1 mL 1 × PBS) twice to remove dead cells, and was resuspended in 0.96 mL of 1 × PBS. Cells were treated with 10 μ L of MilliQ H_2_O or 6.4 mg mL^−1^ SDS in MilliQ H_2_O, and then with 30 μ L of 1 mM nitrocefin. Samples were mixed by rapid inversion, and 0.2 mL of each biological replicate was aliquoted in triplicate into a flat-bottomed 96-well plate (Corning, Corning, NY, USA). Plates were incubated in an Epoch 2 microplate spectrophotometer (BioTek, Winooski, VT, USA) at 30 °C with continuous shaking (237 rpm), and OD_492_ readings were recorded automatically every 15 min for 18 h. Mean blank-corrected peak OD_492_ values were used to quantify fold differences in periplasmic nitrocefin hydrolysis, an established proxy for OM permeability, relative to cell-free 1 × PBS controls.

### Outer membrane potential assays

OM potential assays were performed using cytochrome C as described previously (Revilla-Guarinos et al., 2013; Menon et al., 2022). Briefly, 1 mL of bacterial suspension adjusted to 5.0 × 10^9^ CFU mL^−1^ was centrifuged (5,000 *g*, 2 min), resuspended in 0.97 1 × PBS, and treated with 30 μ L of 5 mg mL^−1^ cytochrome C. Samples were mixed gently by end-over-end inversion, incubated at RT for 10 min, and centrifuged (3,500 *g*, 2 min) to pellet cells and adsorbed cytochrome C. 0.2 mL of each biological replicate was aliquoted in triplicate into a flat-bottomed 96-well plate (Corning, Corning, NY, USA), and single-timepoint OD_530_ readings were collected using an Epoch 2 microplate spectrophotometer (BioTek, Winooski, VT, USA). Mean blank-corrected OD_530_ values were used to quantify fold differences in supernatant cytochrome C concentration relative to cell-free 1 × PBS controls. Mean values significantly lower and higher than 1.0 were considered indicative of decreased and increased OM potential, respectively.

### Serum survival assays

Bacterial cultures were adjusted to 1 × 10^5^ CFU mL^−1^ in LB Lennox containing 85% v/v normal human serum (NHS) (BioIVT, Westbury, NY, USA), and were incubated statically at 30 °C for 4 h. After 1, 2, and 4 h of incubation, 0.2 mL samples were collected, serially diluted in LB Lennox, and 0.1 mL of each dilution was spread-plated on LB Lennox 1.5% w/v agar plates. 0.1 mL of uninoculated 85% v/v NHS LB Lennox was plated to verify serum sterility. Plates were incubated statically at 30 °C for 48 h, and colony counts were used to calculate viable CFUs at each timepoint.

### Growth inhibition assays

Growth inhibition assays with antimicrobial compounds (Supplementary Table S3) were performed and analyzed using methods described previously (Weber et al., 2020; Lauman et al., 2026), with minor modifications. Briefly, 20 µL of bacterial culture, adjusted to 10^6^ CFU mL^−1^ in LB Lennox, was added to a flat-bottomed 96-well plate (Corning, Corning, NY, USA) and combined with 0.16 mL of LB Lennox and 20 µL of antimicrobial compounds at 2-fold increasing dilutions ranging from 20 to 2,560 µg mL^−1^, resulting in final concentrations of 2–256 µg mL^−1^. SDS and colistin were additionally tested at 2-fold concentrations up to 2,048 and 4,096 µg mL^−1^, respectively. Plates were incubated at 30 °C, shaking at 225 rpm, for 48 h, and OD_600_ was measured immediately before (*t_i_*) and after (*t_f_*) incubation. Corrected growth (*t_f_* - *t_i_*) was normalized to the mean of untreated (0 µg mL^−1^) control wells and expressed as percent growth, while percent growth inhibition was calculated as 100% growth. Subinhibitory and inhibitory concentrations were defined as the lowest twofold concentrations at which bacterial growth was reduced by 50% (Half-Maximal Inhibitory Concentration [IC_50_]) and 95% (IC_95_), respectively, relative to untreated controls.

### Phage-antibiotic synergy assays

Synergy assays with KS12 and colistin were conducted and analyzed as described previously (Lauman and Dennis, 2023; Lauman et al., 2026), with minor modifications. Briefly, 0.1 mL of bacterial culture, adjusted to 10^6^ CFU mL^−1^ in LB Lennox, was aliquoted into flat-bottomed 48-well plates (Corning, Corning, NY, USA). Treatment mixtures (0.2 mL total volume; 0.1 mL per component) were then added to establish the following conditions: (i) KS12 alone (5 × 10^6^ PFU mL^−1^ in SM, balanced with MilliQ H_2_O), (ii) colistin alone (5,120 µg mL^−1^ in MilliQ H_2_O, balanced with SM), (iii) KS12 (5 × 10^6^ PFU mL^−1^ in SM) combined with colistin (5,120 µg mL^−1^ in MilliQ H_2_O), (iv) KS12 alone (5 × 10^6^ PFU mL^−1^ in SM, balanced with MilliQ H_2_O; for delayed colistin treatment), or (v) colistin alone (5,120 µg mL^−1^ in MilliQ H_2_O, balanced with SM; for delayed KS12 treatment). After a 10 min incubation at RT, 0.7 mL of LB Lennox was added per well. Final concentrations at *t* = 0 h were 512 µg mL^−1^ colistin, 1 × 10^5^ CFU mL^−1^ bacteria, and 5 × 10^5^ PFU mL^−1^ KS12 (MOI = 5). At *t* = 24 h, an additional 0.1 mL was added to all wells. For simultaneous-treatment conditions (i-iii), wells received a 1:1 mixture of SM and MilliQ H_2_O. In conditions (iv), wells received colistin (5,120 µg mL^−1^ in MilliQ H_2_O), while in condition (v) wells received KS12 (5 × 10^6^ PFU mL^−1^ in SM), thereby producing staggered treatment conditions.

Plates were incubated for 48 h at 30 °C with shaking (225 rpm), and 1 mL was subsequently extracted from each well, subjected to three centrifugation-wash cycles (12,000 *g*, 5 min, 0.5 mL LB Lennox) to remove antibacterial agents, and resuspended in 1 mL LB Lennox. Resuspended cells were serially diluted, and 0.1 mL aliquots of each dilution were spread-plated onto LB Lennox 1.5% w/v agar plates, which were incubated statically at 30 °C for 48 h. Colony counts were used to calculate viable CFUs at endpoint, and endpoint GRCs were computed to evaluate phage-antibiotic synergy (PAS) as described previously mathematically (Lauman et al., 2026).

### Phage stability assays

In serum stability assays, KS12 was diluted in SM or SM containing 85% v/v NHS (BioIVT, Westbury, NY, USA) to 1 × 10^7^ PFU mL^−1^, and was incubated statically at 4 °C, 30 °C, or 37 °C for 48 h. 0.1 mL samples were collected after 24 and 48 h, and were serially diluted in SM, and titers were verified using the double-layer agar method (Kamal and Dennis, 2015).

In hemolymph stability assays, larvae were anesthetized on ice and injected with 10 μ L of KS12 diluted in 1 × PBS to 5 × 10^8^ PFU mL^−1^ (*in vivo* titer ≈ 10^8^ PFU mL^−1^ of hemolymph), and larvae were incubated statically at 30 °C or 37 °C for 48 h. At 24 and 48 h, larvae were decapitated with sterile razors and the hemolymph of larvae in biological replicate groups (*n* = 5) was pooled, serially diluted in SM, and titers were verified using the double-layer agar method.

In macrophage stability assays, KS12 was diluted to 3 × 10^6^ PFU mL^−1^ in a 1:5 mixture of SM and 5% FBS-supplemented DMEM (Cytiva, Marlborough, MA, USA) containing 3 × 10^5^ confluent RAW 264.7 macrophages (phage-to-cell ratio [P: C] ≈ 10), and mixtures were incubated in a flat-bottomed 24-well plate (Corning, Corning, NY, USA) at 37 °C, with 5% CO_2_, for 24 h. After 24 h, 0.1 mL samples were collected, centrifuged (350 *g*, 5 min) to pellet macrophages, and supernatants were serially diluted in SM, and titers were verified using the double-layer agar method. Macrophage-free mixtures were tested to control for the effects of FBS-supplemented DMEM.

KS12 titers were normalized to the initial ( $t_{0}$ ) titers and expressed as % stability at each timepoint for all medium and temperature conditions.

### Endotoxin removal and quantification

Endotoxin removal from crude KS12 lysate was performed using three serial passages through an EndoTrap column (EndoTrap HD 5/1 Column kit; LIONEX GmbH, Germany), following the protocol provided by the manufacturer, and the preparation was subsequently sterilized using a 0.45 μ m MCE filter (Merck Millipore, Ireland) and stored at 4 °C. Quantification of endotoxin in crude and endotoxin-reduced KS12 preparations, as well as heat-killed *B. cenocepacia* K56-2 and sterile SM, was conducted using a Pierce Chromogenic Endotoxin Quant kit (ThermoFisher Scientific, Waltham, MA, USA) following the protocol provided by the manufacturer. Endotoxin-reduced KS12 preparations and re-titers were verified on *B. cenocepacia* K56-2 following endotoxin removal. Endotoxin-rich and endotoxin-reduced preparations were then adjusted to equivalent phage titers, resulting in endotoxin concentrations that spanned a biologically relevant range of macrophage stimulation.

### Macrophage stimulation assays

Exposure assays with RAW 264.7 macrophages were conducted as described previously (Lillico et al., 2023), with minor modifications. Briefly, cells were resuspended in 0.5 mL of conditioned DMEM and seeded into a flat-bottomed 24-well plate (Corning, Corning NY) to a density of 3 × 10^5^ macrophages per well. Plates were incubated at 37 °C, with 5% CO_2_, for 24 h to permit cell attachment, and wells were then rinsed with 0.5 mL 1 × PBS and filled with 0.4 mL 5% FBS-supplemented DMEM. Macrophages were then given 0.1 mL of treatment components to achieve the following exposure conditions: (i) heat-killed *B. cenocepacia* K56-2 (KS12-free; [LPS] = 6.85 × 10^4^ ng mL^−1^, dose = 6.85 × 10^3^ ng), (ii) endotoxin-rich KS12 preparation ([KS12] = 10^7^ PFU mL^−1^, P: C ratio ≈ 35; [LPS] = 125 ng mL^−1^, dose = 12.5 ng), (iii) endotoxin-reduced KS12 preparation ([KS12] = 10^7^ PFU mL^−1^, P: C ratio ≈ 35; [LPS] = 25 ng mL^−1^, dose = 2.5 ng), or (iv) SM (KS12-free; [LPS] < 0.01 ng mL^−1^). Plates were incubated at 37 °C in 5% CO_2_ for 24 h.

After incubation, plates were centrifuged (300 *g*, 5 min) to pellet macrophages, and 0.1 mL of supernatant was collected from each well and submitted to Eve Technologies (Calgary, Canada) for multiplex cytokine analysis using the Mouse Cytokine Pro-inflammatory Focused 10-Plex Discovery Assay. Production of specific cytokines by RAW 264.7 cells was deemed biologically insignificant if the concentration was < 10 pg. mL^−1^ in all treatment groups.

0.1 mL of the remaining supernatants was aliquoted in triplicate into flat-bottomed 96-well plates (Corning), combined with 50 μ L of 2.5% phosphoric acid containing 1% sulphanilamide and 0.01% naphthyl ethylenediamine dihydrochloride (NED), and incubated at RT in the dark for 7 min. Plates were centrifuged (300 *g*, 5 min) and absorbance at 570 nm (A_570_) readings were collected using a Synergy H1 microplate reader (BioTek, Winooski, VT). Plate-specific A_570_-nitrite standard curves were generated using sodium nitrite and were used to estimate the concentration of nitrite, an established proxy for the concentration of unstable ROS of nitric oxide (NO), in each well (Gagioti et al., 2000; Lillico et al., 2023).

Cytokine and nitrite concentrations were log-transformed prior to statistical analysis using log(x + c), where c was defined as one-half of the lower limit of detection (LLOD/2), to accommodate values near or below the detection threshold and improve normality.

### Statistical analysis

Statistical details of experiments are provided in figure legends and, where necessary, in experiment-specific methods sections. N denotes independent biological replicates derived from independently grown bacterial cultures, macrophage passages, or independent *G. mellonella* infection cohorts. Where applicable, n refers to the number of individual *G. mellonella* larvae per cohort. Quantitative data are expressed as the mean ± standard error of the mean (SEM) of at least three independent biological replicates. Figures were created with GraphPad Prism v.11.0.0 (GraphPad Software, San Diego, CA, USA), SnapGene v.8.2.1 (Insightful Science, San Diego, CA, USA), and BioRender (BioRender, Toronto, Canada), and data are presented as non-transformed values unless otherwise indicated. All statistical analyses, including Pearson correlations, *t*-tests, one-way analysis of variance (ANOVAs), Tukey’s *post hoc* multiple comparisons tests, and definite integral calculations using the trapezoid approximation method, were conducted using GraphPad Prism v11.0.0 (GraphPad Software). Differences were deemed statistically significant at *p* < 0.05.

## Results

### Myovirus KS12 has therapeutic potential against *B. gladioli*

**Figure 1:**
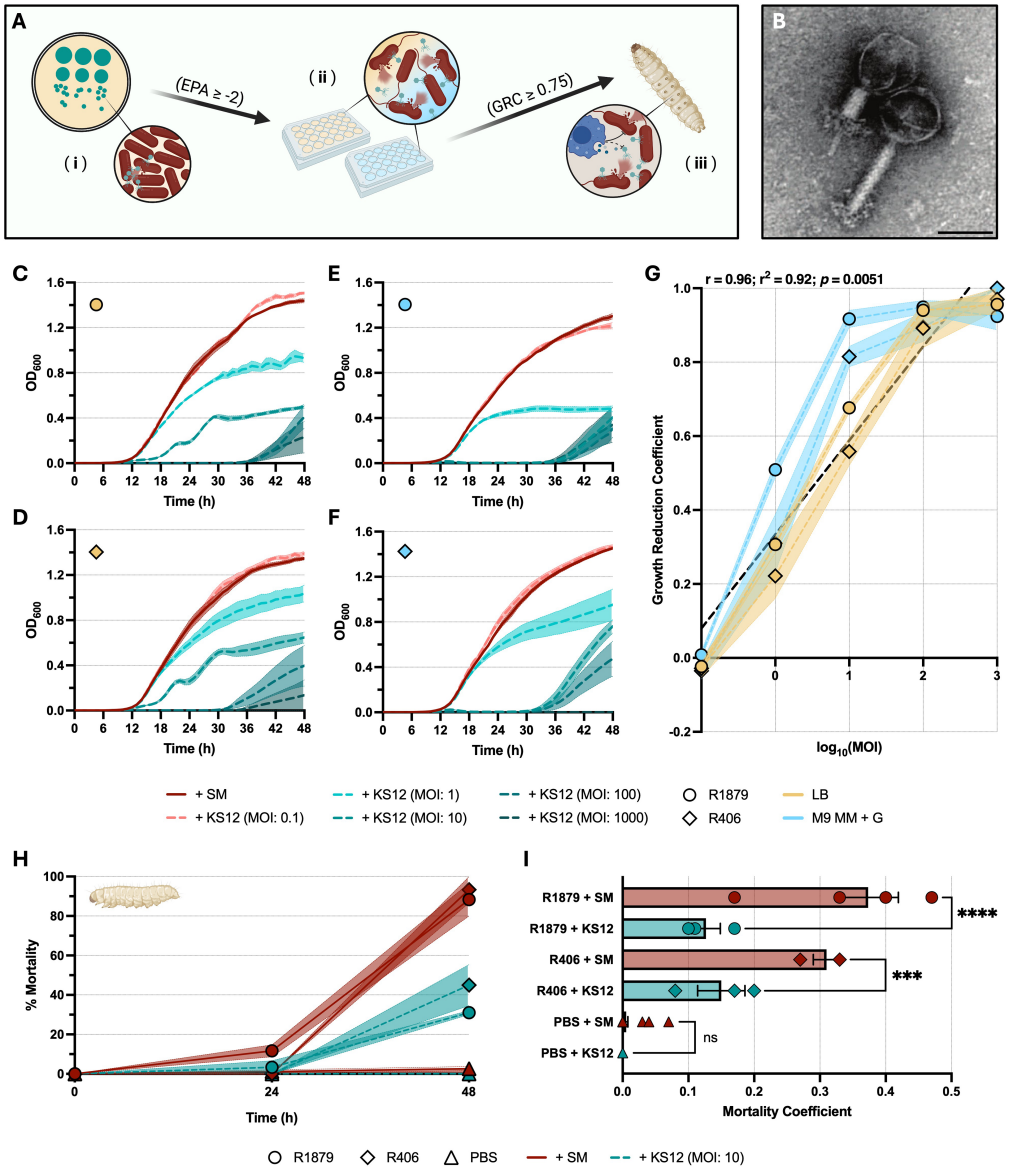
Myovirus KS12 has therapeutic potential against *Burkholderia gladioli.* Schematic of functional phage evaluation pipeline **(A)**: phage-bacterium pairs were screened on solid medium (i), pairings satisfying EPA ≥ –2 were subsequently tested for planktonic killing ability (ii) in LB Lennox (yellow) or glucose-supplemented M9 MM (blue), and pairings that also satisfied GRC ≥ 0.75 on at least one of these media was finally tested in larvae of *Galleria mellonella* (iii). Transmission electron micrograph of KS12 virions **(B)**, with the tail tube contracted (left) and extended (right). Reduction in the planktonic density of *B. gladioli* R1879 **(C,E)** and R406 **(D,F)** over time following co-incubation with KS12 (MOIs 10^–1^–10^3^) in LB Lennox **(C,D)** and glucose-supplemented M9 MM **(E,F)**; and GRCs plotted as a function of MOI across all tested conditions **(G)**. Percent mortality **(H)** and mortality coefficient (C_m_; **I**) for larvae of *G. mellonella* infected with R1879 or R406 and treated with KS12 at MOI 10. Legends explaining the symbol and color coding are provided below the relevant panels. Scale bar in **B** represents 100 nm. Lines **(C–F)**, points **(G,H)**, and bars **(I)** depict the means of at least three biological replicates, shown as points in **(I)**, while shaded regions **(C–H)** and error bars **(I)** represent SEM of those replicates. Statistical differences between groups in **(I)** were computed using a one-way ANOVA followed by Tukey’s *post hoc* multiple-comparisons tests, with ****, ***, and ns (no significant differences), respectively, indicating *p* < 0.0001, *p* < 0.001, and *p* > 0.05. Heavy dashes in **(G)** trace the best-fit line, for which the Pearson correlation coefficient (*r*), coefficient of determination (*r*^2^), and significance of slope deviation from zero (p) are provided.

In an effort to identify therapeutic candidates against *B. gladioli*, we developed a rapid, tripartite *in vitro*–*in vivo* screening pipeline in which phages scoring above pre-defined performance thresholds advanced to subsequent stages (Figure 1A). Phages were progressively screened for infectivity, efficiency of activity on solid medium, and planktonic growth reduction capacity in rich and minimal media, and phages satisfying all *in vitro* thresholds were then examined for their capacity to reduce mortality in a *G. mellonella* infection and rescue model. In screening 10 partially characterized *Burkholderia* phages (Supplementary Table S2) against 12 isolates of *B. gladioli* (Supplementary Table S1), we observed infectivity in 40 (33.3%) phage-host combinations, but only 15 (12.5%) pairs satisfied the efficiency of phage activity (EPA) threshold, EPA ≥ –2 (Supplementary Figure S1). A mere five (4.2%) combinations satisfied the growth reduction coefficient (GRC) threshold, GRC ≥ 0.75 at one or more multiplicities of infections (MOIs) across tested media (Supplementary Figure S2), and all five of these phages reduced *in vivo* mortality coefficients (*C_m_*) by at least 50% relative to untreated controls (Supplementary Figure S3). Curiously, two phage-host pairs with universally low GRCs, tested *in vivo* to form an outgroup, increased *C_m_* in *G. mellonella* relative to untreated controls. Indeed, *in vivo* mortality reduction correlated strongly with GRCs in rich (*r* = 0.89; *r*^2^ = 0.78; *p* = 0.008; Supplementary Figure S4A) and minimal (*r* = 0.93; *r*^2^ = 0.87; *p* = 0.002; Supplementary Figure S4B) media, but not with EPA on solid medium (Supplementary Figure S4C), validating our tripartite approach. Although several phages in our library satisfied our proposed *in vitro* and *in vivo* performance thresholds, the strongest antibacterial effects were exhibited by the largely uncharacterized *Burkholderia* phage KS12.

KS12 is an FL bacteriophage of the myovirus morphotype (capsid diameter: 102.2 ± 2.0 nm; tail length: 140.9 ± 2.8 nm; Figure 1B), which forms <1 mm pinprick plaques on all strains within its relatively narrow host range and exhibits maximal plating efficiency on *B. gladioli* clinical isolates R1879 and R406 (Supplementary Figure S1; also see Lauman and Dennis, 2021). KS12 effectively suppressed the planktonic growth of both strains across a range of MOIs in both rich and minimal media (Figures 1C–F), but overall growth suppression capacity correlated strongly with MOI (*r* = 0.96; *r*^2^ = 0.92; *p* = 0.0051; Figure 1G). *In vivo*, treatment with KS12 (MOI = 10) administered 2 h post-infection (hpi) resulted in 2.96-fold and 2.07-fold reductions in the C_m_ of *G. mellonella* larvae infected with R1879 (*p* < 0.0001) and R406 (*p* = 0.0003), respectively, and caused no adverse effects in mock-infected larvae (Figures 1H,I). Taken together, these findings show that KS12 is a promising candidate for treating infections caused by *B. gladioli*.

### KS12 predation selects for survivors with disrupted LPS and permeabilized outer membranes

**Figure 2:**
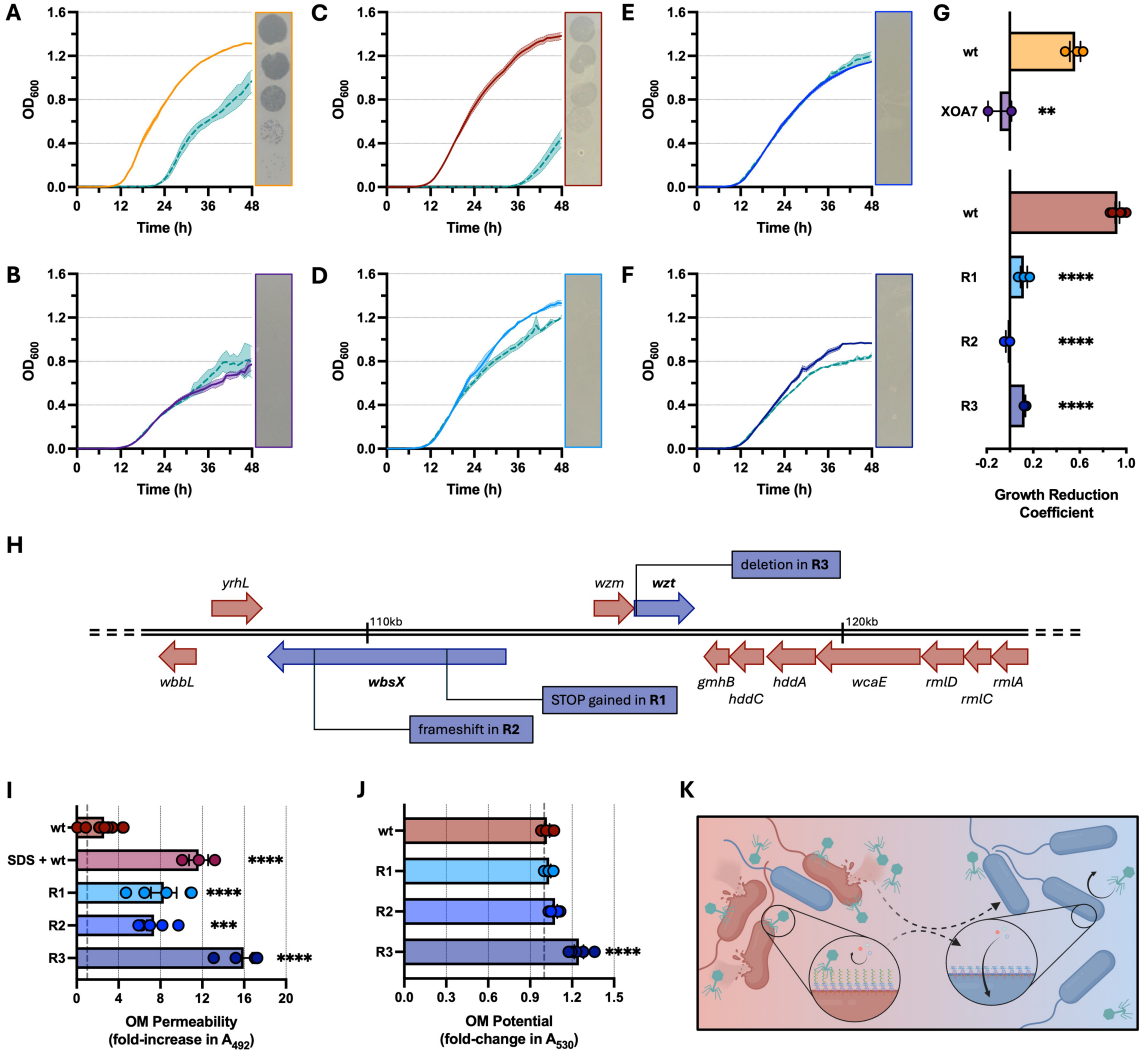
KS12 predation selects for *Burkholderia gladioli* populations with O-antigen defects and permeable outer membranes. Top panels: KS12 plaquing activity (right) and bacterial growth (left) in the presence (teal dashed lines) and absence (solid lines) of KS12 for *Burkholderia cenocepacia* K56-2 wt (**A**, orange) and X0A7 (**B**, purple), and *B. gladioli* R1879 wt (**C**, dark red) and R1 (**D**, light blue), R2 (**E**, blue) and R3 (**F**, dark blue), and the GRCs of KS12 on each of these strains **(G)**. Bottom panels: Genomic map highlighting key mutations in KS12*^R^* isolates of R1879 within the O-antigen biosynthesis and export cluster, with mutated and unaffected genes shown in blue and red, respectively **(H)**. OM permeability **(I)** and electrostatic potential **(J)** of R1879 wt (dark red) and mutants R1-R3 (shades of blue). Lines and bars show the means of at least three biological replicates, which are shown as individual points in panels **(G,I,J)**, while shaded regions and error bars show SEM. Statistical differences between groups were compared using one-way ANOVAs followed by Tukey’s *post hoc* multiple-comparisons tests, with ****, ***, and ** indicating *p* < 0.0001, *p* < 0.001, and *p* < 0.01, respectively. For simplicity, only comparisons with the wt group for each species are shown. Proposed model of KS12 selection for *Burkholderia* populations with permeabilized OM **(K)**, with KS12, wt cells, and KS12*^R^* cells shaded teal, red, and blue, respectively, while solid and dashed arrows respectively depict interactions and the evolutionary trajectory of the bacterial population.

Since the majority of characterized *Burkholderia* phages use lipopolysaccharide (LPS) as a primary receptor (Lauman and Dennis, 2021; Ruest et al., 2023), we next tested whether KS12 is an LPS-binding phage. KS12 infected and suppressed planktonic growth of *B. cenocepacia* K56-2 (Figure 2A), but had no activity against X0A7, a mutant of K56-2 lacking the O-antigen (Figure 2B and Supplementary Table S2). Indeed, the GRC of KS12 was substantially reduced on X0A7 relative to the wild-type (wt) strain (*p* = 0.0065; Figure 2G), indicating that an intact *O*-antigen layer is required for infection and growth suppression by this phage.

To confirm that KS12 uses the same receptor in *B. gladioli*, we recovered three survivor colonies of clinical strain R1879 (R1879- φ KS12-R1, R2, and R3) from independent planktonic killing assays. As expected, KS12 infected and suppressed the growth of the R1879 parent strain (Figure 2C) but lacked infectivity and had significantly reduced GRCs on all three survivor isolates (all *p* < 0.0001; Figures 2D–G). Genomic analyses of these KS12-resistant (KS12^R^) isolates revealed mutations in an O-antigen biosynthesis and export cluster in all three isolates (Figure 2H and Table 1). Isolates R1 and R2 carry nonsense and frameshift mutations, respectively, in the O-antigen glycosyltransferase *wbsX*, with the premature stop in R1 occurring upstream of predicted glycosyltransferase domains and the frameshift in R2 occurring within one of these catalytic domains. In contrast, R3 harbors a six-amino-acid in-frame deletion within the ATP-binding domain of the O-antigen ABC export protein *Wzt*. Because *Wzt* mediates export of assembled O-antigen to the outer membrane (OM), this mutation likely disrupts O-antigen export and results in loss of the surface O-antigen. Consistent with this interpretation, R3 exhibits a severe growth defect relative to its parent strain, roughly analogous to the defect seen in *B. cenocepacia* K56-2 O-antigen-deficient mutant X0A7. In contrast, the growth defects seen in R1 and R2 are comparatively minor (Figures 2A–F). Collectively, these findings suggest that while the O-antigen layers of R1879- φ KS12-R1 and R2 may be aberrant or incomplete, this critical OM structure is likely absent in R3.

**Table 1:** O-Antigen biosynthesis and export-associated mutations observed in KS12^R^ isolates of *Burkholderia gladioli*.

Mutant	Mutation				Predicted effect
	Gene	Protein	Type	Amino acid changes	
R1879- φ KS12-**R1**	*wbsX*	*O*-antigen glycosyltransferase	Nonsense	Arg419 → STOP	Aberrant or truncated O-antigen.
R1879- φ KS12-**R2**	*wbsX*	*O*-antigen glycosyltransferase	Frameshift	Frameshift from Leu1346; STOP inserted at position 1,454.	Aberrant or truncated O-antigen.
R1879- φ KS12-**R3**	*Wzt*	*O*-antigen export ABC transport system ATP-binding protein	In-frame deletion	Δ Ile7-Gly13; Ser inserted at new position 7.	O-antigen totally absent.

As the O-antigen plays a critical role in limiting OM permeability in Gram-negative species (Agrawal and Weisshaar, 2018; Ghai and Ghai, 2018; Cho et al., 2023), we next examined whether OM integrity was altered in KS12^**R**^ survivors. Indeed, the OM permeability of R1879- φ KS12-R3 was increased 6.1-fold relative to the R1879 wt strain (*p* < 0.0001), and was 1.9 and 2.2-fold higher than those of isolates R1 and R2, respectively (both *p* < 0.0001). Notably, permeability in R3 also exceeded that of the wt strain artificially permeabilized with a sub-inhibitory dose of sodium dodecyl sulfate (SDS) by 1.4-fold (*p* = 0.0257). In contrast, the OMs of R1 and R2 were less permeable than that of the SDS-treated parent strain, and exhibited only 3.2-fold (*p* < 0.0001) and 2.8-fold (*p* = 0.0006) increases in permeability relative to the unpermeabilized wt strain (Figure 2I). Collectively, these results indicate that *wbsX* mutants R1 and R2 exhibit milder OM permeabilization phenotypes than the *Wzt* mutant R3, consistent with the predicted effects of these mutations on O-antigen structure. Although Menon et al. recently reported decreased OM potentials in LPS-defective mutants of *Pseudomonas aeruginosa* (Menon et al., 2022), we instead observed a modest 1.2-fold (*p* < 0.0001) increase in OM potential in R3 relative to the parent strain (Figure 2J), possibly reflecting exposure of positively charged L-Ara4N moieties normally shielded by the O-antigen in the LPS outer leaflet (Mahenthiralingam et al., 2005; Hollaus et al., 2014). In contrast, membrane potentials in *wbsX* mutants R1 and R2 remained unchanged. Taken together, these findings suggest that predation by KS12 selects for *B. gladioli* populations with altered or absent O-antigen layers and consequently permeabilized OMs (Figure 2K).

### KS12^R^ survivors exhibit attenuated virulence and hypersensitivity to serum and antimicrobials

**Figure 3:**
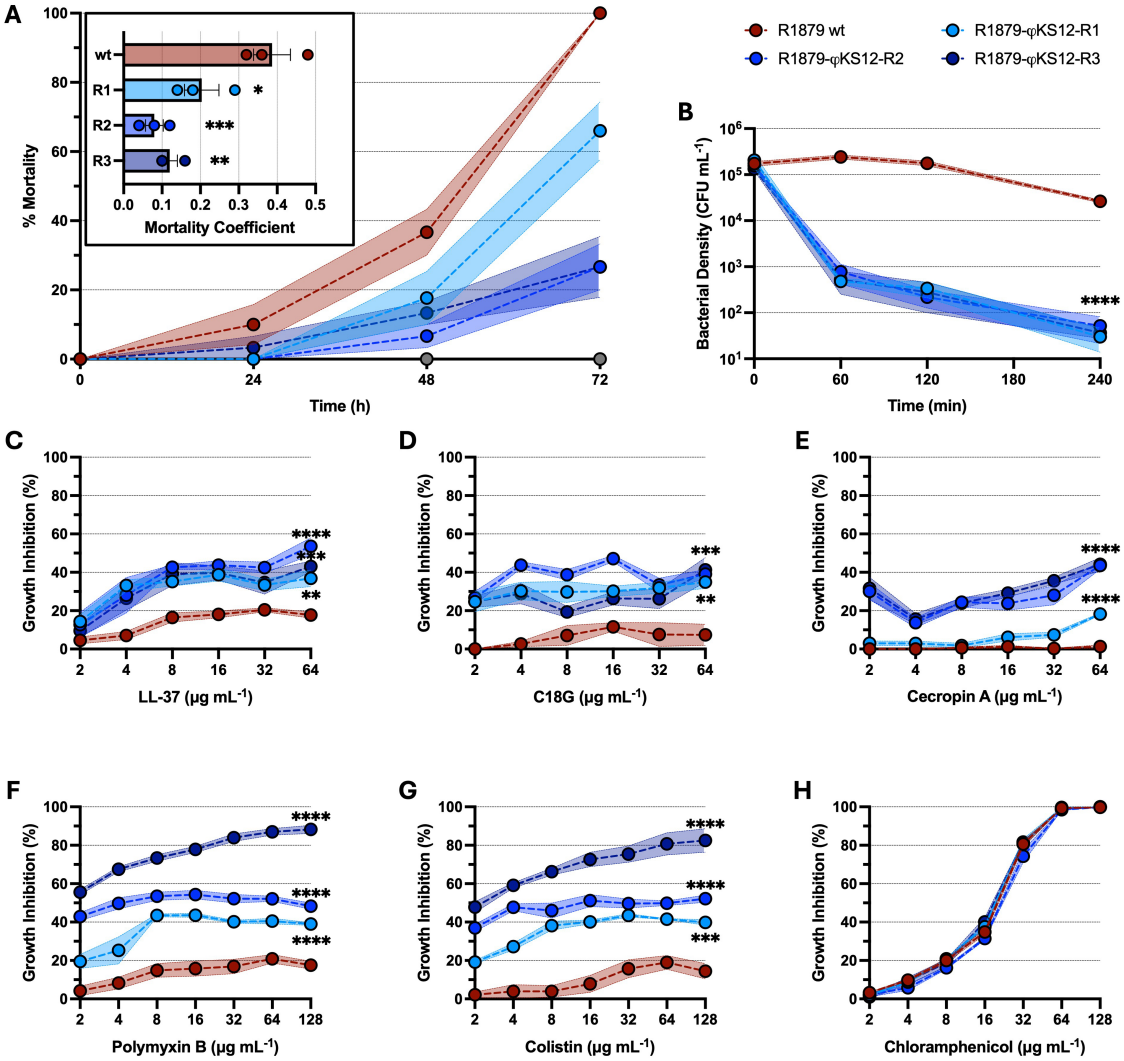
KS12^R^ mutants are attenuated for virulence and hypersensitive to serum and cationic antimicrobials. Percent mortality over time (main) and mortality coefficients (C_m_; inset) of *Galleria mellonella* larvae infected with wt R1879 (dark red) or KS12*^R^* mutants R1 (light blue), R2 (blue), or R3 (dark blue; **A**), and viable cell count over time for these strains in NHS **(B)**. Growth inhibition, expressed as the percent reduction in growth relative to untreated controls, for wt and KS12*^R^* mutants of R1879 treated with antimicrobial peptides LL-37 **(C)**, C18G **(D)**, and cecropin A **(E)**, and antibiotics polymyxin B **(F)**, colistin **(G)**, and chloramphenicol **(H)**. Bars and points show the means of at least three biological replicates, which are additionally shown as individual points in the inset of panel **(A)**, while shaded regions show SEM of these replicates. Statistical differences between R1879 wt and KS12*^R^* mutants were compared using one-way ANOVAs followed by Dunnett’s *post hoc* multiple-comparisons tests, with ****, ***, **, and * indicating *p* < 0.0001, *p* < 0.001, *p* < 0.01, and *p* < 0.05, respectively. For simplicity, only comparisons at the maximal timepoint or concentration are shown in panels **(B–H)**; note that the significance indicator in panel **(B)** is valid for all three mutants, while the top indicators in panels **(D,E)** are valid for both mutants R2 and R3.

Since LPS truncation is often associated with decreased virulence and increased sensitivity to certain antimicrobials (Gao et al., 2022; Menon et al., 2022; Zulk et al., 2022; Ruest et al., 2023), we next explored whether our KS12^**R**^ isolates exhibit these phenotypes. Indeed, the *C_m_* of *G. mellonella* infected with isolates R1879- φ KS12-R1, R2, and R3 were 1.9-fold (*p* = 0.0181), 4.8-fold (*p* = 0.0009), and 3.2-fold (*p* = 0.0021) lower relative to larvae infected with the wt strain (Figure 3A), and over 70% of larvae inoculated with R2 and R3 remained alive 72 hpi. As well, viable cell density following incubation in normal human serum (NHS) was, on average, 2.83 log-fold lower among KS12^R^ isolates compared to their parent strain (all *p* < 0.0001; Figure 3B), with viable cell density approaching the lower limit of detection among all three isolates. Finally, percent growth inhibition was significantly increased at the highest doses of antimicrobial peptides LL-37 (R1: 2.1-fold, *p* = 0.0036; R2: 3.0-fold, *p* < 0.0001; R3: 2.5-fold, *p* = 0.0003; Figure 3C), C18G (R1: 4.7-fold, *p* = 0.0026; R2: 5.3-fold, *p* = 0.0008; R3: 5.6-fold, *p* = 0.0004; Figure 3D), and Cecropin A (R1: 14.0-fold; R2: 33.5-fold; R3: 34.0-fold; all *p* < 0.0001; Figure 3E) in all three isolates relative to R1879 wt. Collectively, these findings suggest that KS12^**R**^ survivors exhibit attenuated virulence and hypersensitivity to human serum and cationic antimicrobial peptides associated with both human and arthropod humoral immunity (Supplementary Table S3).

Similar to members of the Bcc (Zhou et al., 2007; Segonds et al., 2009), *B. gladioli* strains exhibit sensitivity to certain antibiotics but are intrinsically resistant to polymyxins such as colistin (Supplementary Table S4). LPS mutants of *B. cenocepacia* are known to exhibit increased polymyxin sensitivity (Ruest et al., 2023), we therefore queried whether our KS12^**R**^ mutants exhibit such sensitization. Indeed, percent growth inhibition was increased significantly at the highest doses of polymyxin B (R1: 2.2-fold; R2: 2.8-fold; R3: 5.0-fold; all *p* < 0.0001; Figure 3F) and colistin (R1: 2.8-fold, *p* = 0.0007; R2: 3.6-fold, *p* < 0.0001; R3: 5.7-fold, *p* < 0.0001; Figure 3G) in all three isolates relative to the R1879 wt. *Wzt* mutant R1879- φ KS12-R3 additionally exhibited modestly increased percent growth inhibition by 2048 µg mL^−1^ SDS (1.3-fold, *p* = 0.0003; Supplementary Figure S5A), 16 µg mL^−1^ benzalkonium chloride (1.5-fold, *p* < 0.0001; Supplementary Figure S5B) and 32 µg mL^−1^ ceftazidime (1.5-fold, *p* = 0.0017; Supplementary Figure S5C), relative to its wt parent. Together, these data indicate that KS12^R^ survivors exhibit increased susceptibility to membrane-targeting antibiotics whose efficacy depends on OM permeability. Notably, we observed no increases in growth inhibition by chloramphenicol (Figure 3H), consistent with chloramphenicol susceptibility being primarily governed by efflux and enzymatic inactivation rather than OM permeability (Schwarz et al., 2004).

### Synergy between KS12 and colistin decimates *Burkholderia* populations

**Figure 4:**
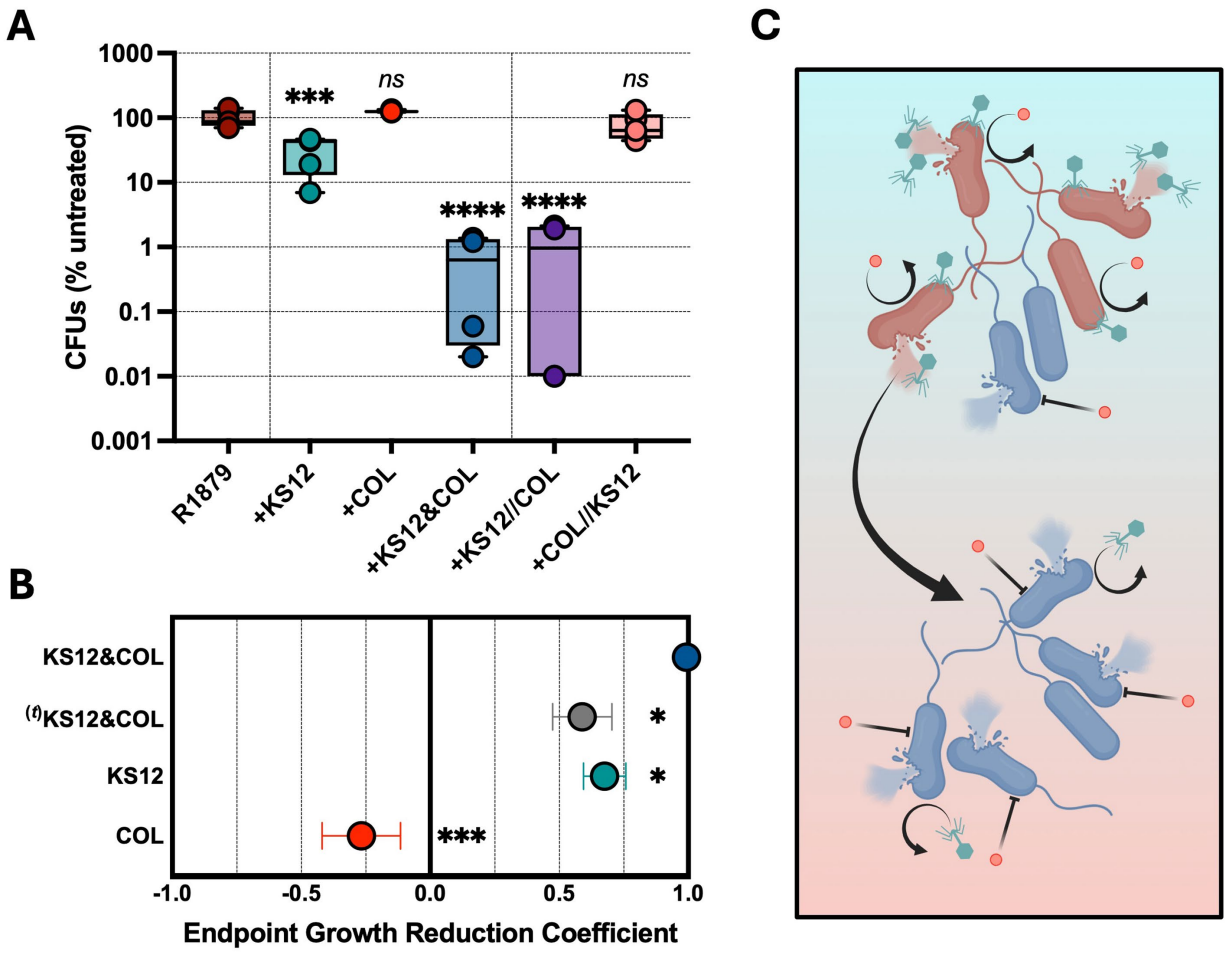
KS12 synergizes with colistin to decimate *Burkholderia gladioli* populations. **(A)** Viable cell count, expressed as percentage of untreated controls, for *Burkholderia gladioli* R1879 treated with SM (mock; dark red), single-timepoint treatments with KS12 (teal), colistin (bright red), and both agents applied simultaneously (dark blue), and staggered treatment with either KS12 (purple) or colistin (light red) applied first. **(B)** Endpoint cell count-derived GRCs for all single-timepoint treatment groups, with gray representing the theoretical value expected for a mathematically additive antibacterial effect. Black bars and points in panels **(A,B)**, respectively, show the medians and means of at least five biological replicates, which are shown as individual points in panel **(A)**, while error bars in panel **(B)** show SEM. Statistical differences between groups were compared using one-way ANOVAs followed by Tukey’s *post hoc* multiple-comparisons tests, with ****, ***, *, and ns (no significant differences) indicating *p* < 0.0001, *p* < 0.001, *p* < 0.05, and *p* > 0.05, respectively. For simplicity, only comparisons with the mock-treatment and simultaneous combination-treatment groups are shown in panels **(A,B)**, respectively. **(C)** Proposed mechanism of KS12-colistin synergy, with wt and KS12*^R^* R1879 cells shown in dark red and blue, respectively. KS12 and colistin are shown in teal and bright red, respectively, while small and large black arrows depict interactions and the evolutionary trajectory of the bacterial population.

Since predation by KS12 selects for subpopulations sensitized to colistin, we next probed whether these agents interact synergistically to reduce bacterial populations. Indeed, although the phage reduced viable colony-forming units (CFUs) of R1879 only 0.49 log-fold (*p* = 0.0007) after 48 h when used alone, simultaneous co-administration with an otherwise ineffective dose of colistin reduced CFUs 2.18 log-fold (*p* < 0.0001). Staggered treatment was ineffective when KS12 was applied 24 h after colistin but was as effective as simultaneous co-administration when KS12 was applied first, and reduced viable CFUs 1.99 log-fold (*p* < 0.0001) relative to untreated R1879 (Figure 4A). Moreover, the empirical CFU-based endpoint GRC of the simultaneous KS12-colistin combination was substantially higher not only than those of KS12 (*p* = 0.018) and colistin (*p* = 0.0002) on their own, but also compared to the theoretical value expected if the antibacterial effects of these agents were merely additive (*p* = 0.0246), satisfying the mathematical conditions for antibacterial synergism (Figure 4B). Collectively, our results suggest that populations of *B. gladioli* R1879 are decimated by a synergistic antibacterial interaction between KS12 and colistin, wherein KS12 predation eliminates susceptible cells and selects for subpopulations sensitized to colistin (Figure 4C).

### Outer membrane impermeability is a mechanism of colistin resistance

**Figure 5:**
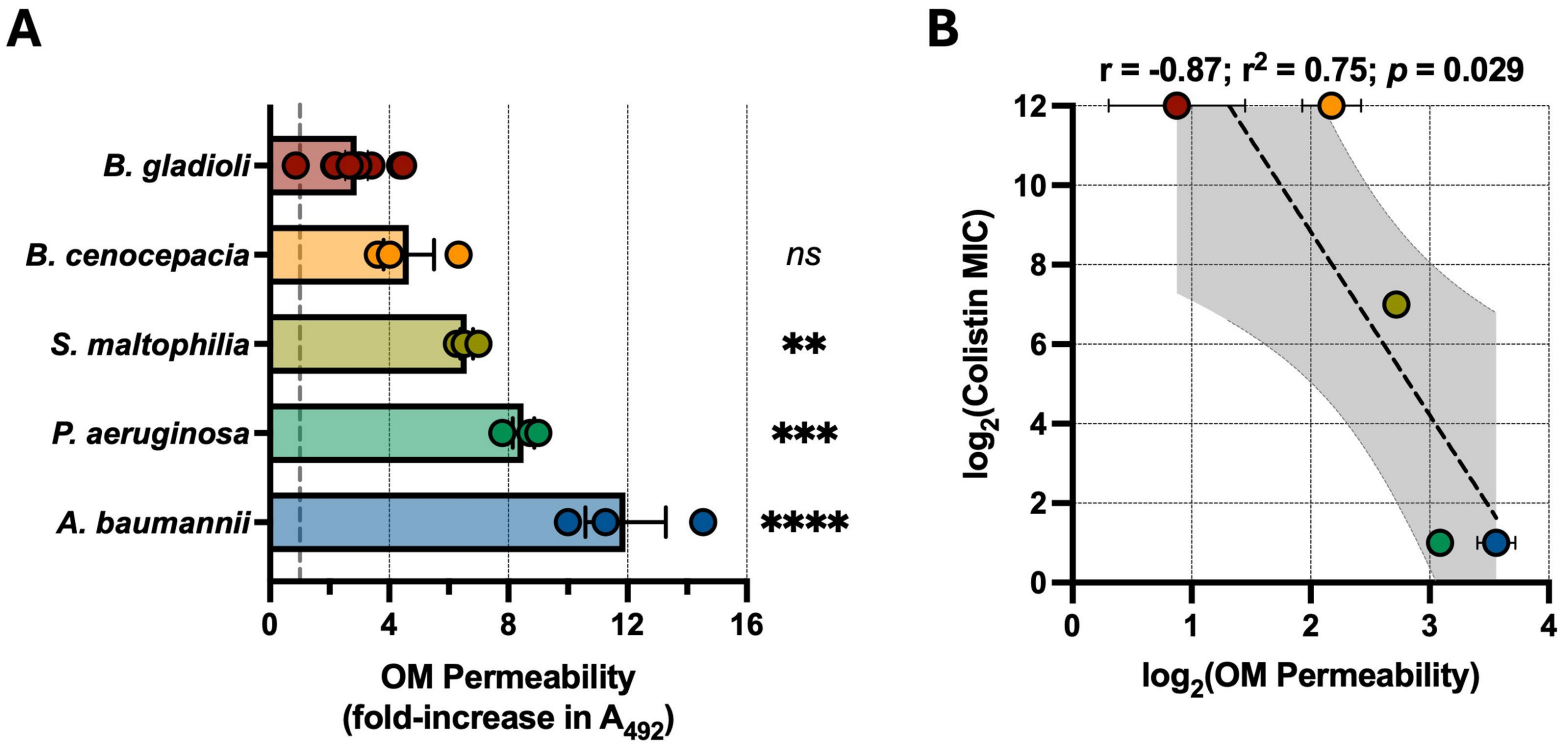
OM permeability correlates inversely with colistin resistance. OM permeability of *Burkholderia gladioli* R1879 (dark red), *Burkholderia cenocepacia* K56-2 (orange), *Stenotrophomonas maltophilia* SMDP92 (asparagus), *Pseudomonas aeruginosa* PA01 (green), and *Acinetobacter baumannii* AB5075 (blue), computed as fold-change in A_492_ (corresponding to periplasmic nitrocefin breakdown) relative to cell-free controls (gray dashed line; **A**), and correlation between log_2_-transformed OM permeability and colistin MICs **(B)**. Bars and points in panels **(A,B)**, respectively, represent the means of at least three biological replicates, shown as points in panel **(A)**, while error bars represent SEM. Statistical differences between *B. gladioli* and other groups in panel **(A)** were compared using a one-way ANOVA followed by Dunnett’s *post hoc* multiple-comparisons tests on log-transformed data, with ****, ***, **, and ns (no significant differences), respectively, indicating *p* < 0.0001, *p* < 0.001, *p* < 0.01, and *p* > 0.05. Heavy dashes in panel **(B)** trace the best-fit line, for which the Pearson correlation coefficient (*r*), coefficient of determination (*r*^2^), and significance of slope deviation from zero (*p*) are provided, and the 90% confidence interval is shaded gray.

To potentially generalize the utility of LPS-binding phages such as KS12, we examined whether OM permeability is associated with colistin sensitivity across other Gram-negative pathogens. Although OM permeability in *B. gladioli* and *B. cenocepacia* was predictably comparable, permeability was 2.3-fold (*p* = 0.0020), 2.9-fold (*p* < 0.0001), and 4.1-fold (*p* < 0.0001) higher in *S. maltophilia*, *P. aeruginosa*, and *A. baumannii*, respectively (Figure 5A), and these pathogens exhibited correspondingly lower colistin minimum inhibitory Concentration (MICs) (Supplementary Table S4). Indeed, we observed a strong inverse correlation between OM permeability and colistin MIC (*r* = −0.87; *r*^2^ = 0.75; *p* = 0.029; Figure 5B). Moreover, *B. cenocepacia* K56-2 *waaL* (X0A7) and *hldA* (Sal1) mutants, deficient in O-antigen and inner core assembly, respectively, displayed 6.4-fold (*p* = 0.0115) and 10.7-fold (*p* = 0.0006) increases in OM permeability relative to wt cells (Supplementary Figure S6), and were previously shown to exhibit substantially reduced colistin MICs (Loutet et al., 2006; Ruest et al., 2023). In contrast, we observed no meaningful correlation between colistin MIC and OM surface potential (Supplementary Table S4 and Supplementary Figure S7), indicating that membrane surface charge plays a comparatively limited role in colistin resistance in these organisms. Taken together, these findings suggest that membrane impermeability may be a mechanism of colistin resistance among these pathogens.

### KS12 is depleted by humoral immunity but interacts minimally with professional phagocytes

**Figure 6:**
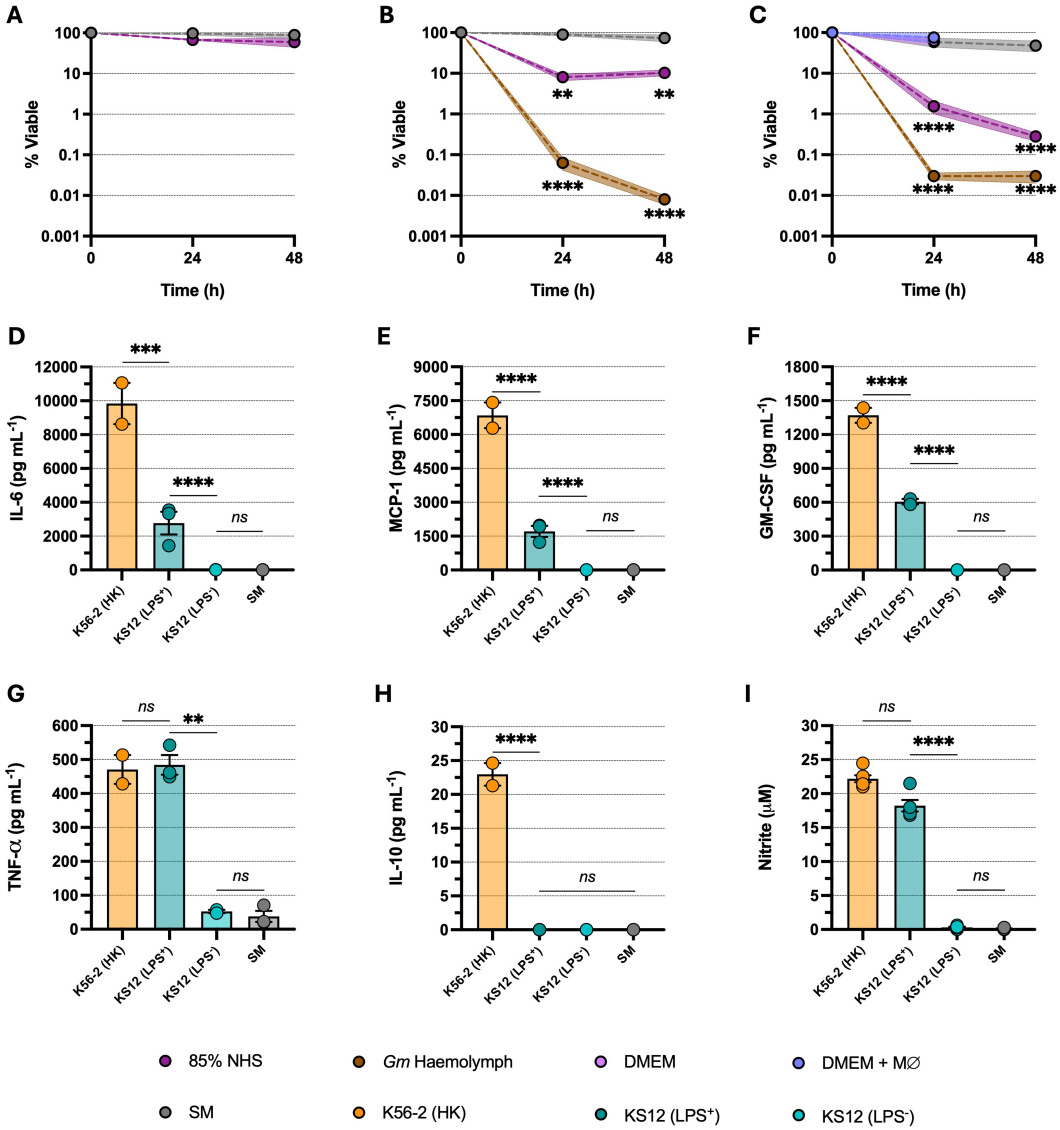
KS12 is depleted by humoral immunity but interacts minimally with macrophages. Top panels: viability of KS12, expressed as percentage of inoculum dose, following incubation in SM (gray), 85% NHS (violet), *Galleria mellonella* hemolymph (brown), and DMEM in the presence (orchid) and absence (lavender) of RAW 264.7 macrophages, at 4°C **(A)**, 30°C **(B)**, and 37°C **(C)**. Bottom panels: pro-inflammatory stimulation of macrophages by SM (gray), heat-killed *Burkholderia cenocepacia* K56-2 (orange), and equal-titer endotoxin-rich (LPS^+^; teal) and endotoxin-reduced (LPS^-^; turquoise) preparations of KS12, measured via production of IL-6 **(D)**, MCP-1 **(E)**, GM-CSF **(F)**, TNF- α **(G)**, IL-10 **(H)**, and nitrite **(I)**. Points and bars in the top and bottom panels, respectively, represent the means of at least three biological replicates, which are shown as open circles in the bottom panels. SEM is depicted via shaded regions and error bars in the top and bottom panels, respectively. Statistical differences between groups were compared using one-way ANOVAs followed by Tukey’s *post hoc* multiple-comparisons tests on log-transformed data, with ****, ***, **, *, and ns (no significant differences) indicating *p* < 0.0001, *p* < 0.001, *p* < 0.01, *p* < 0.05, and *p* > 0.05, respectively. For simplicity, only key comparisons are depicted.

Despite exhibiting *in vivo* efficacy against *B. cenocepacia*, KS12 is known to be highly unstable (Seed and Dennis, 2009; Semler et al., 2014). To determine whether *in vivo* instability is due to inactivation by elements of innate humoral immunity, we compared the viability of KS12 *in vitro* in SM and normalized serum from unprimed human donors (NHS), and *in vivo* in the hemolymph of *G. mellonella*, across a range of temperatures. No statistically or biologically meaningful differences in KS12 viability were observed between SM and NHS at 4 °C (Figure 6A), but viability in NHS was attenuated partially at 30 °C (1.1 log-fold [*p* = 0.0023] and 0.9 log-fold [*p* = 0.0031] reductions at 24 h and 48 h, respectively; Figure 6B) and substantially at 37 °C (1.6 and 2.2 log-fold at 24 h and 48 h, respectively, both *p* < 0.0001; Figure 6C). Viability was also severely compromised in *G. mellonella* hemolymph under all tested conditions (3.2–4.0 log-fold reduction, all *p* < 0.0001; Figures 6B,C), collectively implying that functional KS12 titer decays rapidly when exposed to mammalian or arthropod humoral immunity. Notably, KS12 was significantly less stable in hemolymph relative to NHS at all tested conditions (1.0 log-fold [*p* = 0.0025] by 48 h at 37 °C, 1.7 to 3.1 log-fold [all *p* < 0.0001] at other timepoints; Figures 6B,C), suggesting that either *G. mellonella* hemolymph is more potent than NHS, or KS12 inactivation by humoral immunity is not the only contributing factor *in vivo*.

To identify whether breakdown by professional phagocytes, such as macrophages, might contribute to KS12 instability, we exposed the phage to RAW 264.7 murine macrophages at a phage-to-cell ratio (P:C) of 10. No significant differences were observed in the viability of KS12 exposed to macrophages, cell-free DMEM, or SM after 24 h of incubation at 37 °C (Figure 6C), suggesting that these phagocytes do not irreversibly internalize or degrade KS12 in this context.

To probe our hypothesis that the association between instability and antibacterial efficacy among certain phages could be driven by phage-mediated activation of pro-inflammatory innate immune responses, we next assessed the ability of KS12 to stimulate pro-inflammatory responses in RAW 264.7 murine macrophages. Macrophages were exposed to equal-titer doses of endotoxin-rich and endotoxin-reduced KS12 preparations, along with heat-killed *B. cenocepacia* and SM, and supernatants were assayed for pro-inflammatory cytokines and metabolites. Critically, no significant differences were observed between the immunostimulatory capacities of SM and endotoxin-reduced KS12 preparations (Figures 6D–I). Relative to endotoxin-rich preparations, endotoxin-reduced KS12 triggered non-zero but significantly decreased production of interleukin 6 (IL-6) (2.73 log-fold, *p* < 0.0001; Figure 6D), nitrite (1.77 log-fold, *p* < 0.0001; Figure 6I) and tumor necrosis factor-alpha (TNF- α) (0.96 log-fold, *p* = 0.0035; Figure 6G). Monocyte chemoattractant protein-1 (MCP-1) and granulocyte-macrophage colony-stimulating factor (GM-CSF) production was absent in macrophages exposed to endotoxin-reduced preparations, but was significantly higher (both *p* < 0.0001; Figures 6E,F) among cells treated with endotoxin-rich KS12. Collectively, these findings suggest that in the absence of endotoxin, KS12 particles alone are insufficient to trigger pro-inflammatory responses.

No significant differences were observed between the capacities of heat-killed *B. cenocepacia* and endotoxin-rich KS12 preparations to trigger production of TNF- α (Figure 6G) and nitrite (Figure 6I). Conversely, heat-killed *B. cenocepacia* stimulated significantly higher production of IL-6 (*p* = 0.0003; Figure 6D), MCP-1 (*p* < 0.0001; Figure 6E), GM-CSF (*p* < 0.0001; Figure 6F), and IL-10 (*p* < 0.0001; Figure 6H), although increases in the observed concentrations of these cytokines were relatively low, and IL-10 production was stimulated only by *B. cenocepacia*, collectively suggesting that higher doses of endotoxin or additional PAMPs are required to trigger maximal production of these cytokines. Production of IL-1 β , IL-2, IFN- γ , IL-4, and IL-12p70 was assessed, but detected concentrations of these cytokines were below the threshold of biological significance (Supplementary Figure S8).

Taken together, these findings indicate that although humoral innate immune factors readily inactivate KS12, phage particles themselves appear to interact minimally with professional phagocytes and do not independently trigger pro-inflammatory responses.

## Discussion

Therapeutic bacteriophages targeting *B. gladioli* remain poorly characterized, and no studies have systematically examined the antibacterial efficacy, antivirulence steering potential, or immunogenicity of phages infecting this emerging opportunistic pathogen. Here we show that the LPS-binding FL myovirus KS12 suppresses *B. gladioli* growth *in vitro* and infection-associated mortality *in vivo* (Figure 1) while selecting for resistant subpopulations with altered or absent O-antigen layers (Figure 2 and Table 1). These changes compromise OM integrity (Figure 2), producing bacterial populations that are attenuated for virulence and highly susceptible to serum, cationic antimicrobial peptides, and polymyxins (Figure 3). Consistent with this evolutionary trade-off, KS12 and colistin interact synergistically to reduce bacterial densities dramatically (Figure 4). These observations are consistent with our broader finding that OM permeability correlates strongly with colistin susceptibility across multiple Gram-negative pathogens (Figure 5). Although we initially speculated that phage-mediated immune stimulation could contribute to bacterial clearance, our results instead indicate that therapeutic efficacy arises primarily from direct bactericidal activity and antivirulence steering rather than from direct immunostimulation by phage particles (Figure 6).

Myovirus KS12 emerged as the strongest candidate in our phage panel and exhibited robust antibacterial activity against *B. gladioli* across both *in vitro* and *in vivo* systems. Suppression of planktonic bacterial growth by KS12 was observed across a range of conditions and strongly correlated with MOI, reinforcing findings from previous systematic studies of phages infecting *Burkholderia* species and other pathogens (Jansen et al., 2018; Taha et al., 2018; Storms et al., 2020; Lauman and Dennis, 2023; Supina et al., 2025). Even at an intermediate MOI, KS12 treatment significantly reduced mortality in *G. mellonella* larvae infected with *B. gladioli*, echoing previous studies with this and other *Burkholderia* phages targeting Bcc member *B. cenocepacia* (Seed and Dennis, 2009; Carmody et al., 2010; Lynch et al., 2013; Semler et al., 2014; Roszniowski et al., 2017). Notably, *in vivo* protection correlated strongly with reduced bacterial growth in liquid culture, suggesting that planktonic killing assays may serve as a useful early indicator of *in vivo* antibacterial efficacy. Conversely, while plating metrics are useful for establishing host range and disqualifying phage-host combinations with poor infectivity (Haines et al., 2021; Lauman and Dennis, 2023), they do not appear to predict antibacterial performance *in vivo*. Together, these observations reinforce the value of integrating plating analyses, liquid culture assays, and *in vivo* infection models into early-stage phage evaluation pipelines and identify KS12 as a potential therapeutic candidate against *B. gladioli*.

Resistance to phage predation, and thus to phage therapy, frequently arises through modification or loss of cell-surface structures that serve as phage attachment sites, and such alterations can impose fitness costs on bacteria in specific contexts (Labrie et al., 2010; Oechslin, 2018; Lauman and Dennis, 2021). In Gram-negative organisms, the LPS layer plays a critical role in maintaining OM integrity and resistance to antibiotics and immune effectors such as antimicrobial peptides, suggesting that LPS-targeting phages may provide an effective means of steering bacterial populations toward more clinically tractable phenotypes (Loutet et al., 2006; Loutet and Valvano, 2011; Agrawal and Weisshaar, 2018; Cho et al., 2023; Ruest et al., 2023). Consistent with this paradigm, KS12 selects for *B. gladioli* subpopulations in which the O-antigen layer is absent or altered, resulting in compromised OM integrity, reduced virulence *in vivo*, and hypersensitivity to serum components, antimicrobial peptides, and polymyxins. Taken together, these phenotypes are consistent with a common mechanism in which disruption of the O-antigen compromises OM barrier function, simultaneously increasing vulnerability to host immune effectors and membrane-targeting antibiotics. The differing phenotypes observed among KS12-insensitive isolates are consistent with the predicted functional consequences of identified mutations in the O-antigen export pathway. While disruption of the O-antigen export protein Wzt likely abolishes surface O-antigen entirely, mutations in the glycosyltransferase WbsX may instead produce incomplete or aberrant O-antigen structures, resulting in comparatively milder OM defects. Similar evolutionary trade-offs have been reported for LPS-binding phages infecting several pathogenic species, including *B. cenocepacia*, *Escherichia coli*, *P. aeruginosa*, and *Salmonella enterica* (Gao et al., 2022; Menon et al., 2022; Zulk et al., 2022; Ruest et al., 2023). In line with these observations, our results suggest that OM impermeability could represent a mechanism of resistance to polymyxins such as colistin, and antivirulence steering with LPS-binding phages could therefore serve as a broadly exploitable therapeutic strategy for sensitizing Gram-negative pathogens to antibiotics of last resort. Although combined treatment with KS12 and colistin did not fully eradicate *B. gladioli* populations *in vitro*, the observed >99% reduction in bacterial density suggests that such combinations could substantially facilitate pathogen clearance by host immune defenses *in vivo*, particularly since surviving subpopulations exhibit impaired resistance to host immune effectors. In this context, phage steering may impose an evolutionary trap in which escape from phage predation increases susceptibility to host immune effectors and membrane-targeting antibiotics. Together, these findings illustrate how evolutionary pressures imposed by therapeutic phages can be leveraged to steer bacterial populations toward sensitivity to both antibiotics and host immune defenses, thereby permitting exploitation through combination therapies.

In addition to shaping bacterial evolution, phages may influence therapeutic outcomes by modulating host immune responses. Phages are increasingly recognized as immunostimulatory in at least some contexts (Shepardson et al., 2017; Krut and Bekeredjian-Ding, 2018; Gogokhia et al., 2019; Podlacha et al., 2024; Le et al., 2025), and we therefore speculated that phage-mediated activation of non-specific innate immune responses could contribute to the simultaneous depletion of both phages and bacteria observed in previous studies (Seed and Dennis, 2009; Semler et al., 2014; Lauman and Dennis, 2021). However, KS12 virions themselves did not measurably stimulate pro-inflammatory responses in RAW 264.7 macrophages and did not appear to be degraded or irreversibly internalized by these cells, suggesting minimal direct interaction with at least this line of professional phagocytes. These observations are consistent with some reports on phage immunogenicity but contrast with others, underscoring the variability and ongoing debate surrounding phage-immune interactions (Miernikiewicz et al., 2013; Mirzaei et al., 2016; Shiley et al., 2017; Van Belleghem et al., 2017; Freyberger et al., 2018; Cafora et al., 2021; Le et al., 2025). Although the immunogenicity of phages infecting *Burkholderia* species has received little attention, Carmody et al. reported that virions of the *B. cenocepacia* podovirus BcepIL02 did not measurably enhance inflammatory markers in a murine infection model (Carmody et al., 2010). Importantly, the immunological effects of phage particles themselves must be distinguished from those of phage predation. While purified KS12 particles triggered minimal pro-inflammatory responses, phage-mediated bacterial lysis could nevertheless influence immune signaling indirectly through the release of bacterial antigens such as LPS. Disentangling these effects experimentally remains challenging, however, as bacterial killing by phages and host immune effectors occurs concurrently. Finally, KS12 was rapidly inactivated by both unprimed human serum and *G. mellonella* hemolymph, indicating that humoral innate immunity substantially contributes to phage clearance *in vivo*. Although our findings do not exclude the possibility that opsonized KS12 particles could be internalized by phagocytes in certain tissues (Akira et al., 2006), the data overall suggest that KS12 may interact only minimally with professional phagocytes and has limited intrinsic pro-inflammatory potential, implying that the therapeutic activity of KS12 arises from direct bactericidal activity and antivirulence steering rather than immune activation.

Although KS12 is shown here to be a potentially promising therapeutic candidate against *B. gladioli*, several considerations temper the interpretation of these findings. Critically, genomic characterization of KS12 has remained elusive because extensive DNA modification has thus far prevented sequencing. While functional assays strongly support obligately lytic replication, the absence of a sequenced genome precludes definitive exclusion of lysogenic or otherwise harmful gene content, and genetic characterization therefore remains a major hurdle for the therapeutic deployment of this phage. Moreover, *in vivo* efficacy and immunostimulatory potential were, respectively, evaluated using the *G. mellonella* infection model and the singular RAW 264.7 immortalized cell line, neither of which fully recapitulates the complexities of mammalian infections or immune responses. Future studies in mammalian systems will therefore be necessary to establish the pharmacological and immunological properties of KS12 in more clinically relevant contexts. Finally, the long-term evolutionary dynamics of resistance to KS12 *in vivo* remain unknown. Although the resistant phenotypes observed in this study impose substantial fitness costs and increase sensitivity to immune effectors and antibiotics, the stability of these trade-offs within heterogeneous host environments warrants further investigation. Despite these limitations, the present findings highlight the therapeutic potential of antivirulence phage steering for pathogens exhibiting intrinsic resistance to antimicrobial agents. In particular, the ability of KS12 to select for bacterial subpopulations with compromised OMs reveals a mechanism whereby phage predation can be leveraged to sensitize Gram-negative pathogens to immune effectors and antibiotics of last resort. LPS-targeting phages like KS12 may therefore represent valuable components of combination therapies designed to increase antimicrobial susceptibility and expand treatment options for difficult-to-treat bacterial infections, particularly as future studies clarify how phage-driven selection pressures can be leveraged in clinical settings.

Taken together, our findings suggest that the antibacterial efficacy of KS12 arises primarily from direct bactericidal activity and antivirulence steering rather than from immunostimulation by phage particles. Although KS12 is rapidly inactivated by elements of innate humoral immunity, phage predation and replication during infection are likely sufficient to impose strong selective pressures that reshape bacterial populations. In this phage-host system, predation selects for subpopulations with permeabilized OMs that are attenuated for virulence and highly susceptible to both host immune effectors and polymyxins. These results highlight how evolutionary trade-offs associated with phage resistance can be exploited therapeutically to sensitize bacterial pathogens to antibiotics and immune defenses. More broadly, our study highlights the potential of LPS-targeting phages to act not only as antibacterial agents but also as drivers of antivirulence steering, enabling evolutionary reprogramming of intrinsically resistant pathogens toward clinically tractable phenotypes.

## Data Availability

The original contributions presented in the study are included in the article/Supplementary material, further inquiries can be directed to the corresponding author/s.
