# Supplementary material for: A Burkholderia phage selects for attenuated virulence and antimicrobial hypersensitivity through increased outer membrane permeability
Source: Front Microbiol. 2026 Jul 9;17:1876186. doi: 10.3389/fmicb.2026.1876186 (PMC13391952; doi:10.3389/fmicb.2026.1876186)
Supplement: Supplementary file 1 [file Data_Sheet_1.pdf]

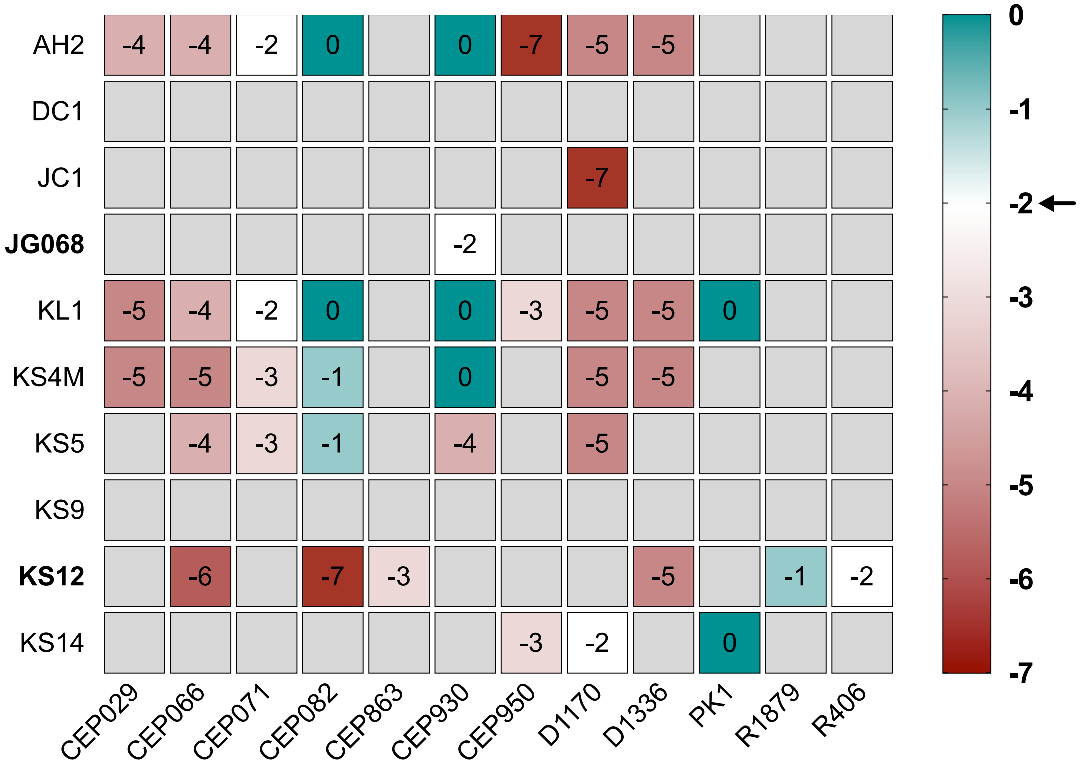

Figure S1: Efficiencies of *Burkholderia* phage activity against *B. gladioli*. EPA scores for partially characterized *Burkholderia* phages (y-axis) targeting strains of *B. gladioli* (x-axis), ranging from a maximum of 0 (teal) to a minimum of -7 (LLOD; dark red), while lack of detectable phage sensitivity is shown in grey. Lytic phages JG068 and KS12 are shown in bold, while all other phages are LC, and the black arrow highlights the threshold of acceptable efficiency of phage activity (EPA $\geq$ -2). Values are representative of at least three biological replicates.

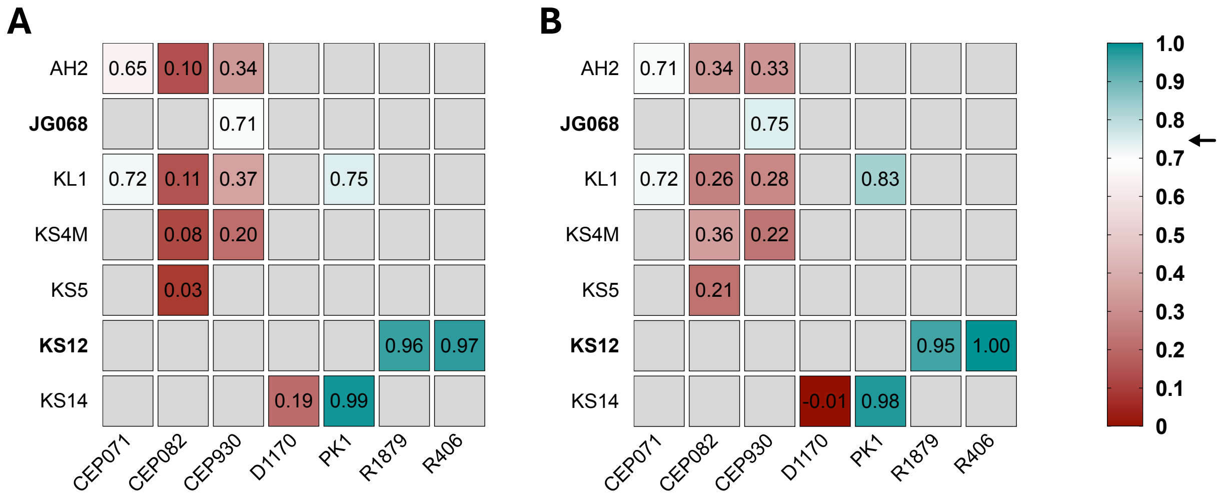

Figure S2: Planktonic killing of *B. gladioli* by *Burkholderia* phages. GRCs of partially characterized *Burkholderia* phages (y-axes) targeting strains of *B. gladioli* (x-axes) at optimal MOIs in LB Lennox (**A**) and glucose-supplemented M9 MM (**B**). Scores range from a maximum of 1 (teal) to an expected minimum of 0 (dark red), while rare negative values are shaded crimson. Grey cells denote phage-host combinations with efficiency of phage activity below the threshold for further testing (i.e. EPA $<$ -2; see **Fig. S1**). For simplicity, only phages and strains for which at least one phage-host pair satisfying EPA $\geq$ -2 was identified are shown. Lytic phages JG068 & KS12 are shown in bold, while all other phages are LC, and the black arrow highlights the threshold of acceptable planktonic growth reduction (GRC $\geq$ 0.75). All values are representative of at least three biological replicates.

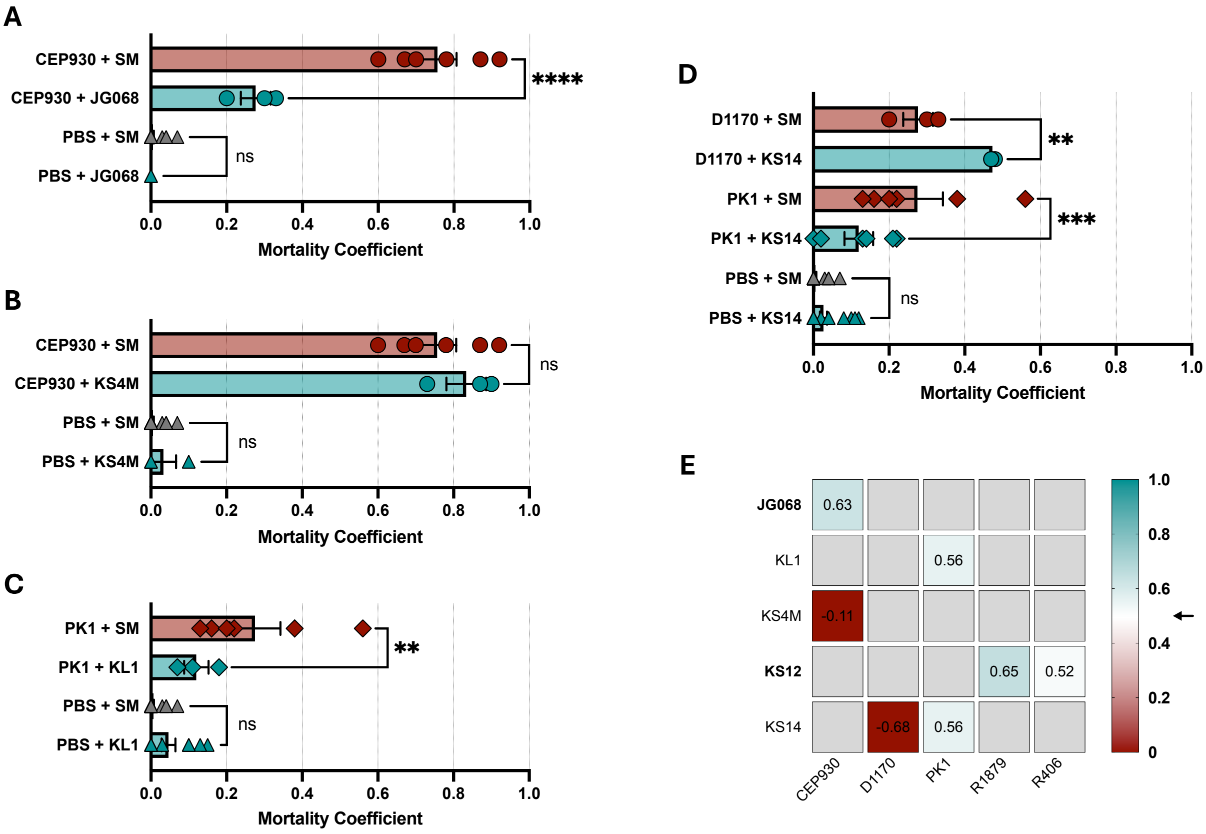

Figure S3: Efficacy of *Burkholderia* phage treatment of *B. gladioli* infections in *G. mellonella*. C_m values for *B. gladioli* infections of *G. mellonella* larvae with or without treatment with *Burkholderia* phages JG068 (**A**), KS4M (**B**), KL1 (**C**), & KS14 (**D**), and **R** C_m values for all phage-host combinations of *G. mellonella* infections investigated in this study (**E**). In panels **A-D**, circles/diamonds and triangles represent infection with indicated *B. gladioli* strains or PBS (mock), respectively, while teal, red and grey respectively indicate treatment with phage, SM following *B. gladioli* infection, and SM following mock infection. Statistical differences between groups in panels **A-D** were compared using one-way ANOVAs followed by Tukey's post-hoc multiple comparisons tests, with ****, ***, **, and *ns* indicating $p < 0.0001$, $p < 0.001$, $p < 0.01$, and $p > 0.05$ respectively. **R** C_m values in panel **E** range from a maximum of 1 (teal) to an expected minimum of 0 (dark red), while negative values are shaded crimson. Grey cells denote phage-host combinations with EPAs or GRCs below the threshold for further testing (i.e. EPA < -2 or GRC < 0.75; see **Figs. S1** & **S2**). For simplicity, only phages and strains for which at least one phage-host pair satisfied EPA ≥ -2 *and* GRC ≥ 0.75 are shown, except for pairs KS4M+CEP930 & KS14+D1170 – which were included to form an outgroup testing the *in vivo* efficacy of phages that perform poorly *in vitro*. In panel **E**, lytic phages JG068 and KS12 are shown in bold, while all other phages are LC, and the black arrow highlights the proposed threshold of acceptable mortality reduction (**R** $C_m \geq 0.50$). Bars in panels **A-D** and values in panel **E** show the means of at least three biological replicates, which are shown as open symbols and for which error bars depict SEM in panels **A-D**.

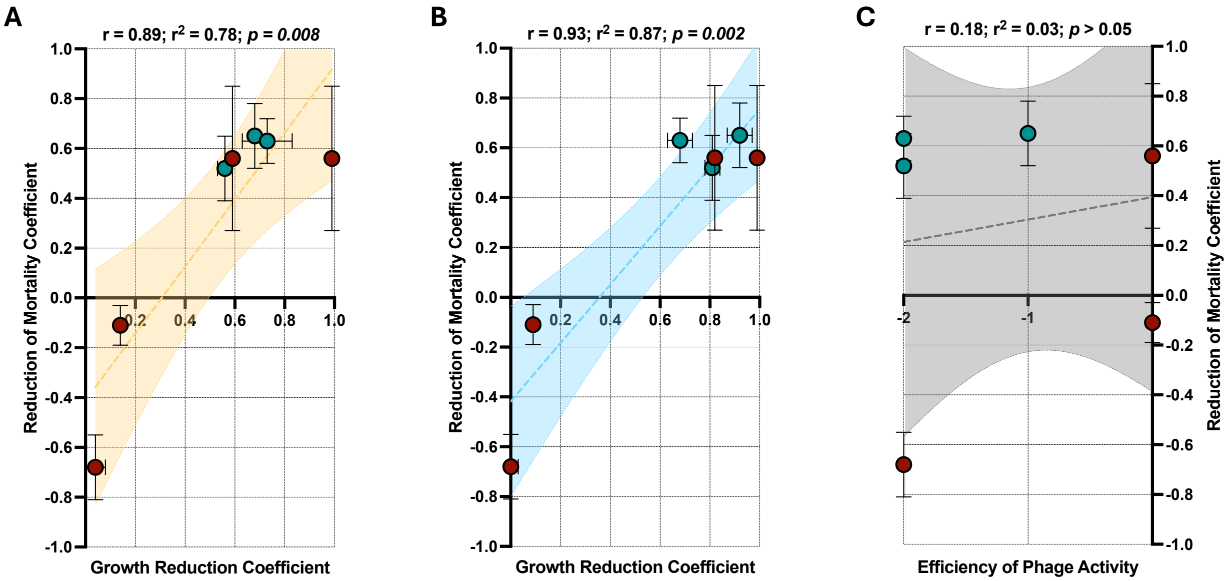

Figure S4: Predictability of *in vivo* mortality reduction using *in vitro* phage efficacy metrics. Correlations between **R** C_m values and GRCs at corresponding MOIs in LB Lennox (**A**) & glucose-supplemented M9 MM (**B**), and EPA (**C**), for all phage-host combinations investigated in *G. mellonella*. Teal and dark red points depict phage-host combinations in which the phages were lytic and LC, respectively. Heavy dashes trace best-fit lines, for which the correlation coefficient (r), coefficient of determination (r^2), and significance of slope deviation from zero (*p*) are provided above each panel, and the 95% confidence intervals of these lines are shown as shaded regions. Points and their error bars show the means and SEM, respectively, of at least three biological replicates.

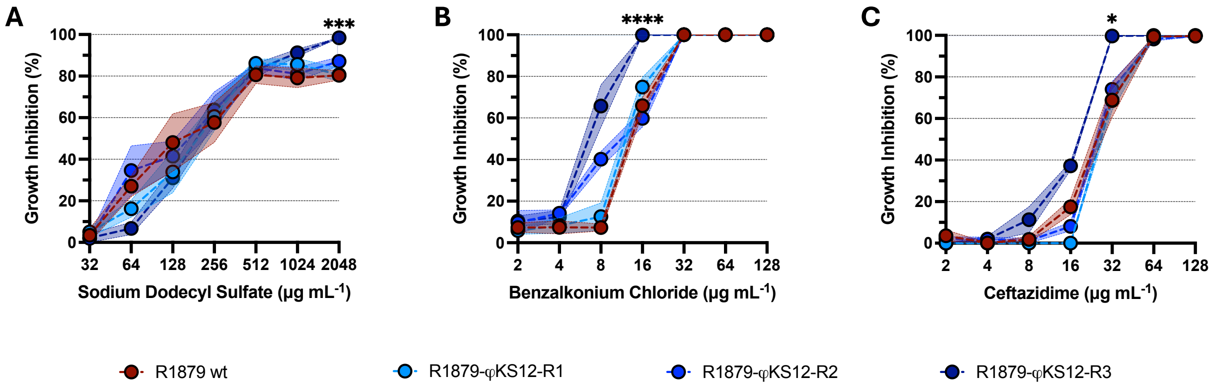

Figure S5: Increased sensitivity unique to hyper-permeable mutant R1879-*φ*KS12-R3. Growth inhibition, expressed as the percent reduction in growth relative to untreated controls, for R1879 wt (red) and KS12^{R} mutants R1 (light blue), R2 (blue) & R3 (dark blue) treated with sodium dodecyl sulfate (**A**), benzalkonium chloride (**B**), and ceftazidime (**C**). Points show the means of at least five biological replicates while shaded regions show SEM. Statistical differences between mutants and the wt strain were compared using one-way ANOVAs followed by Dunnett's post-hoc multiple comparisons tests, with ****, ***, and *, indicating $p < 0.0001$, $p < 0.001$, and $p < 0.05$, respectively. Comparisons between R1879-*φ*KS12-R3 and wt are depicted above relevant compound concentrations, and for simplicity only the most significant differences are shown for each compound.

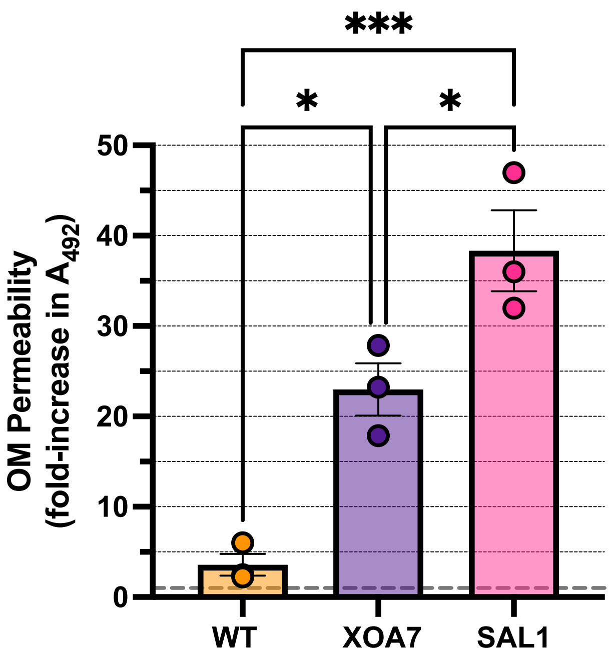

Figure S6: OM permeability of LPS-truncated mutants of *B. cenocepacia* K56-2. OM permeability of *B. cenocepacia* K56-2 wt (orange), O-antigen deficient mutant X0A7 (purple) and LPS inner core-truncated mutant Sal1 (pink), shown as fold-changes in A_{492} relative to cell-free controls (dashed grey line). Bars represent the means of three biological replicates, which are shown as points, while error bars show SEM of those replicates. Statistical differences between groups were compared using one-way ANOVAs followed by Tukey's post-hoc multiple comparisons tests, with *** and * indicating $p < 0.001$ and $p < 0.05$, respectively.

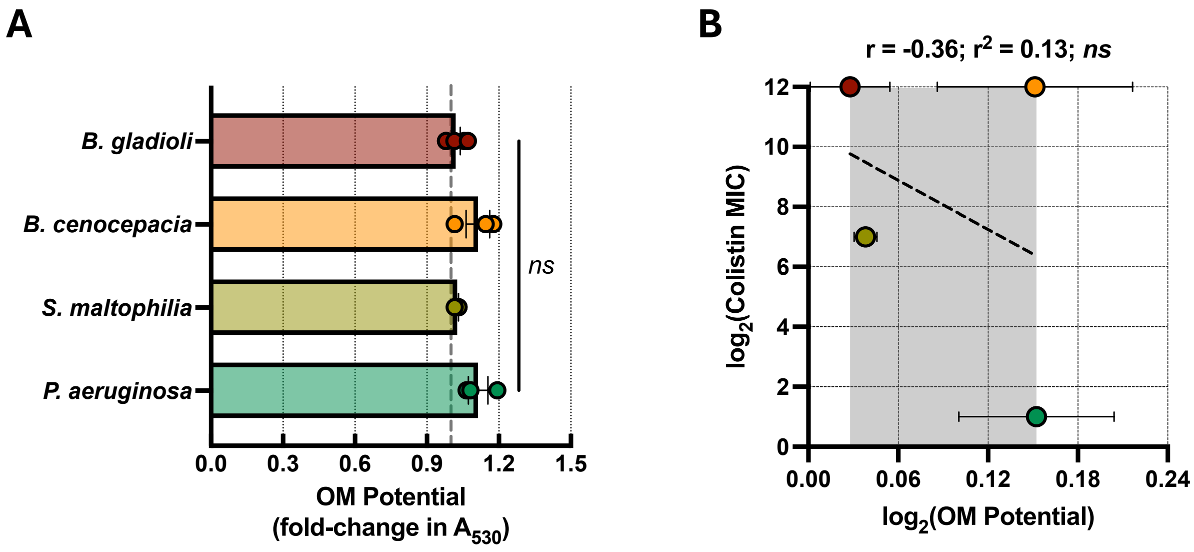

Figure S7: Impact of OM potential on colistin resistance. OM potential of *B. gladioli* R1879 (red), *B. cenocepacia* K56-2 (orange), *S. maltophilia* SMDP92 (asparagus), and *P. aeruginosa* PAO1 (green), computed as fold-change in A_{530} (corresponding to supernatant cytochrome C concentration) relative to cell-free controls (grey dashed line; **A**), and correlation between $\log_2$-transformed OM potential and colistin MICs (**B**). Bars and points in **A** & **B** respectively present the means of at least three biological replicates, shown as points in panel **A**, while error bars show SEM. Statistical differences between groups in **A** were identified using a one-way ANOVA followed by Tukey's post-hoc multiple comparisons tests, with *ns* indicating $p > 0.05$. Heavy dashes in **B** trace the best-fit line, for which the Pearson correlation coefficient (r), coefficient of determination (r^2), and significance of slope deviation from zero (*p*) are provided, and the 90% confidence interval is shaded grey.

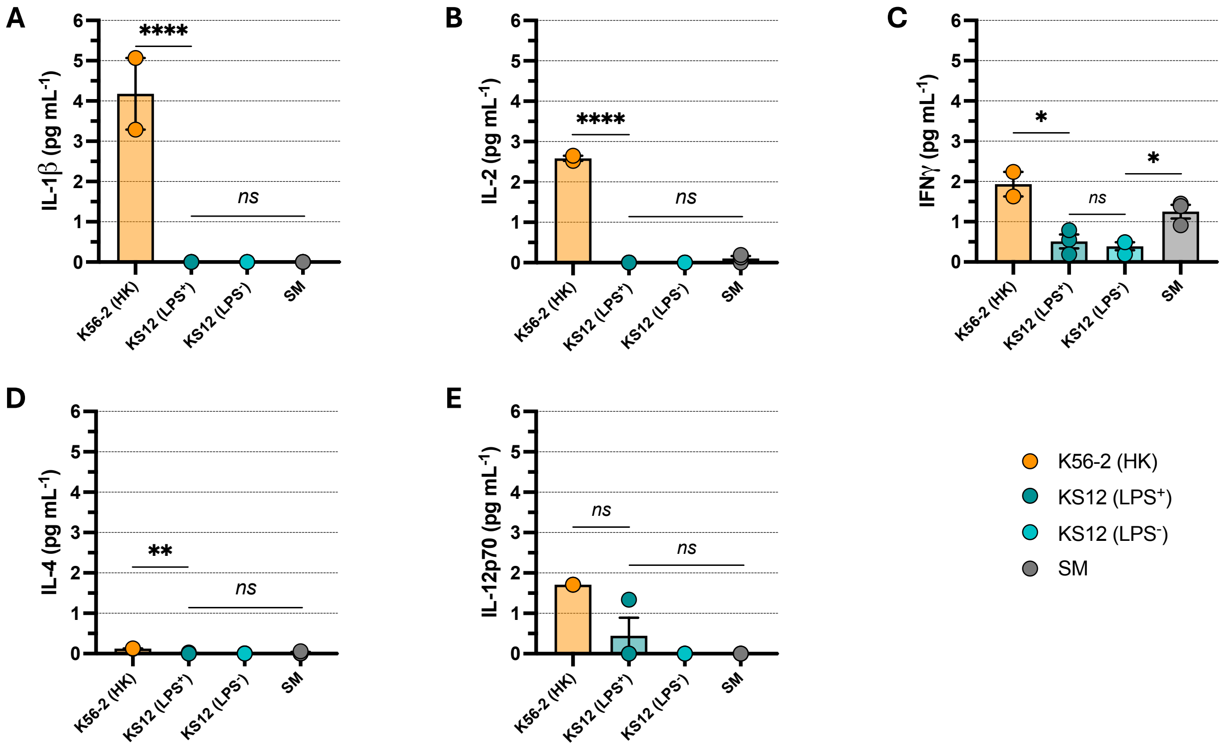

Figure S8: Cytokines for which production is biologically insignificant. Production of IL-1*β* (**A**), IL-2 (**B**), IFN*γ* (**C**), IL-4 (**D**), and IL-12p70 (**E**), all of which are below the threshold of biological significance (< 10 pg ml^{-1} in all groups), by RAW 264.7 murine macrophages stimulated with SM (grey), heat-killed *B. cenocepacia* K56-2 (orange), and equal-titer endotoxin-rich (LPS^+; teal) and endotoxin-reduced (LPS^-; turquoise) stocks of KS12. Bars show the means of three biological replicates, shown as open circles, while error bars depict SEM of these replicates. Statistical differences between groups were compared using one-way ANOVAs followed by Tukey's post-hoc multiple-comparisons tests on log-transformed data, with ****, **, *, and *ns* indicating $p < 0.0001$, $p < 0.01$, $p < 0.05$, and $p > 0.05$ respectively. For simplicity, only key comparisons are depicted.

Table S1: Bacterial strains used in this study.

	Source[1]	Genetic Characteristics		References
		Mutated Gene	Affected Function	
Acinetobacter baumannii				
AB5075	Bone infection, USA			(Jacobs et al., 2014)
Burkholderia cenocepacia				
C6433	CF, Canada			(Seed and Dennis, 2008)
K56-2	CF-e, Canada			(Seed and Dennis, 2008)
K56-2 X0A7	transposon mutant of K56-2	*waaL*	O-antigen biosynthesis	(Ortega et al., 2009)
K56-2 Sal1	transposon mutant of K56-2	*hldA*	LPS inner core biosynthesis	(Loutet et al., 2006)
Van1	CF, Canada			(Davis et al., 2022)
Burkholderia gladioli				
CEP029	CGD, USA			This study
CEP066	Onion, Canada			This study
CEP071	Onion, Brazil			This study
CEP082	Soil, Canada			This study
CEP863	CF, Canada			This study
CEP930	CF, Canada			This study
CEP950	CF, Canada			This study
D1170	CF, Canada			This study
D1336	CF, Canada			This study
PK1	CF, UK			This study
R1879	CF, Canada			This study
R1879-φKS12-R1	$KS12^R$ isolate of R1879	*wbsX*	O-antigen biosynthesis	This study
R1879-φKS12-R2	$KS12^R$ isolate of R1879	*wbsX*	O-antigen biosynthesis	This study
R1879-φKS12-R3	$KS12^R$ isolate of R1879	*wzt*	O-antigen export	This study
R406	CF, Canada			This study
Pseudomonas aeruginosa				
PAO1	Wound, Australia			(Holloway, 1955)
Stenotrophomonas maltophilia				
SMDP92	Lung infection, Brazil			(De Oliveira-Garcia et al., 2002)

[1] Abbreviations: CF: cystic fibrosis; CF-e: spreading endemically among CF patients; CGD: chronic granulomatous disease; $KS12^R$: KS12-resistant.

Table S2: *Burkholderia* phages used in this study.

	Morphology	Source	Receptor	Lifestyle	References
AH2	Siphovirus	*Nandina* sp. soil	Unknown, not LPS	Lysogenization Capable	(Lynch et al., 2012b; Lauman and Dennis, 2023)
DC1	Podovirus	*Dracaena* sp. soil	Unknown.	Lysogenization Capable	(Lynch et al., 2012a; Lauman and Dennis, 2023)
JC1	Podovirus	*Geranium & Petunia* sp. soil	LPS inner core	Lysogenization Capable	(Davis et al., 2022)
JG608	Podovirus	Sewage	LPS O-antigen	Obligately Lytic	(Lynch et al., 2013; Lauman and Dennis, 2023; Ruest et al., 2023)
KL1	Siphovirus	Sewage	Unknown, not LPS	Lysogenization Capable	(Lynch et al., 2012b; Lauman and Dennis, 2023)
KS4M	Myovirus	Mutant of DK4 / BcepMu / KS4	LPS O-antigen	Lysogenization Capable	(Seed and Dennis, 2005; Lauman and Dennis, 2023; Ruest et al., 2023)
KS5	Myovirus	Onion rhizosphere	LPS inner core	Lysogenization Capable	(Seed and Dennis, 2005; Lynch et al., 2010b; Lauman and Dennis, 2023; Ruest et al., 2023)
KS9	Siphovirus	Lysogen of *B. pyrrocinia*	LPS O-antigen	Lysogenization Capable	(Seed and Dennis, 2005; Lynch et al., 2010a; Lauman and Dennis, 2023; Ruest et al., 2023)
KS12	Myovirus	*Dietes* sp. soil	LPS O-antigen	Functionally Lytic	(This Study; Seed and Dennis, 2008)
KS14	Myovirus	*Dracaena* sp. soil	Unknown	Lysogenization Capable	(Lynch et al., 2010b; Lauman and Dennis, 2023)

Table S3: Antimicrobial compounds used in this study.

Antibiotics	Manufacturer	Identifier	Concentration Range (μg/mL)	Class / Type
Ceftazidime	Sigma-Aldrich	CAS: 72558-82-8	2 - 256	cephalosporin
Chloramphenicol	Sigma-Aldrich	CAS: 56-75-7	2 - 256	--
Ciprofloxacin	Sigma-Aldrich	CAS: 85721-33-1	2 - 256	fluoroquinolone
Colistin	MP Biomedicals	CAT: 194157	2 - 4096	polymyxin
Meropenem	Sigma-Aldrich	CAS: 119478-56-7	2 - 256	β-lactam
Minocycline	Sigma-Aldrich	CAS: 13614-98-7	2 - 256	tetracycline
Polymyxin B	Sigma-Aldrich	CAS: 1405-20-5	2 - 256	polymyxin
Antimicrobial Peptides				
C18G	Anaspec	CAT: AS-62412	2 - 64	Human AMP
Cecropin A	Anaspec	CAT: AS-24009	2 - 64	Insect AMP
LL-37	Anaspec	CAT: AS-61302	2 - 64	Human AMP
Surfactants				
Benzalkonium Chloride	Sigma-Aldrich	CAS: 63449-41-2	2 - 256	detergent
Sodium Dodecyl Sulfate	ThermoFisher	CAS: 151-21-3	2 - 2048	detergent

Table S4: Minimum inhibitory concentrations (MICs[1]; in μg/mL) of select antibiotics on relevant gram-negative bacteria[2].

	**Meropenem**		**Minocycline**		**Ciprofloxacin**		**Chloramphenicol**		**Ceftazidime**		**Colistin**	
	MIC_{50}	MIC_{95}	MIC_{50}	MIC_{95}	MIC_{50}	MIC_{95}	MIC_{50}	MIC_{95}	MIC_{50}	MIC_{95}	MIC_{50}	MIC_{95}
Acinetobacter baumannii												
AB5075[‡]											2	2
Burkholderia cenocepacia												
K56-2[‡]											> 4096	> 4096
Burkholderia gladioli												
CEP082	0.5	1	4	4	2	2	64	64	32	64	> 256	> 256
CEP930	2	8	16	32	4	4	128	128	128	256	> 256	> 256
D1170	1	4	8	32	32	64	64	128	64	128	> 256	> 256
PK1	1	1	4	8	16	16	32	64	32	64	> 256	> 256
R1879[‡]	1	4	4	4	2	2	32	64	32	64	> 4096	> 4096
R406	1	4	2	4	2	2	32	64	64	128	> 256	> 256
Pseudomonas aeruginosa												
PA01[‡]											2	2
Stenotrophomonas maltophilia												
SMDP92[‡]											256	256

[1] MIC_{50} and MIC_{95} correspond to the minimum log_2-spaced concentrations required to reduce planktonic growth by 50% and 95%, respectively.

[2] All antibiotics were tested at log_2-spaced concentrations in the range 2-256 μg/mL except meropenem, which was tested between 0.5-64 μg/mL. In strains of particular interest ([‡]), colistin was tested at log_2-spaced concentrations up to 4096 μg/mL, which approaches the solubility limit in water.

Supplemental References:

Davis, C. M., Ruest, M. K., Cole, J. H., and Dennis, J. J. (2022). The Isolation and Characterization of a Broad Host Range Bcep22-like Podovirus JC1. *Viruses* 14, 1–34. doi: 10.3390/v14050938

De Oliveira-Garcia, D., Dall'Agnol, M., Rosales, M., Azzuz, A. C. G. S., Martinez, M. B., and Girón, J. A. (2002). Characterization of flagella produced by clinical strains of Stenotrophomonas maltophilia. *Emerg. Infect. Dis.* 8, 918–923. doi: 10.3201/eid0809.010535

Holloway, B. W. (1955). Genetic recombination in Pseudomonas aeruginosa. *J. Gen. Microbiol.* 13, 572–581. doi: 10.1099/00221287-13-3-572

Jacobs, A. C., Thompson, M. G., Black, C. C., Kessler, J. L., Clark, L. P., McQueary, C. N., et al. (2014). AB5075, a highly virulent isolate of acinetobacter baumannii, as a model strain for the evaluation of pathogenesis and antimicrobial treatments. *mBio* 5. doi: 10.1128/mBio.01076-14

Lauman, P., and Dennis, J. J. (2023). Synergistic Interactions among Burkholderia cepacia Complex-Targeting Phages Reveal a Novel Therapeutic Role for Lysogenization-Capable Phages . *Microbiol. Spectr.* 11. doi: 10.1128/spectrum.04430-22

Loutet, S. A., Flannagan, R. S., Kooi, C., Sokol, P. A., and Valvano, M. A. (2006). A complete lipopolysaccharide inner core oligosaccharide is required for resistance of Burkholderia cenocepacia to antimicrobial peptides and bacterial survival in vivo. *J. Bacteriol.* 188, 2073–2080. doi: 10.1128/JB.188.6.2073-2080.2006

Lynch, K. H., Abdu, A. H., Schobert, M., and Dennis, J. J. (2013). Genomic characterization of JG068, a novel virulent podovirus active against Burkholderia cenocepacia. *BMC Genomics* 14. doi: 10.1186/1471-2164-14-574

Lynch, K. H., Seed, K. D., Stothard, P., and Dennis, J. J. (2010a). Inactivation of Burkholderia cepacia complex phage KS9 gp41 identifies the phage repressor and generates lytic virions. *J. Virol.* 84, 1276–1288. doi: 10.1128/JVI.01843-09

Lynch, K. H., Stothard, P., and Dennis, J. J. (2010b). Genomic analysis and relatedness of P2-like phages of the Burkholderia cepacia complex. *BMC Genomics* 11, 599. doi: 10.1186/1471-2164-11-599

Lynch, K. H., Stothard, P., and Dennis, J. J. (2012a). Characterization of DC1, a broad-host-range Bcep22-like podovirus. *Appl. Environ. Microbiol.* 78, 889–891. doi: 10.1128/AEM.07097-11

Lynch, K. H., Stothard, P., and Dennis, J. J. (2012b). Comparative analysis of two phenotypically-similar but genomically-distinct Burkholderia cenocepacia -specific bacteriophages. *BMC Genomics* 12.

Ortega, X., Silipo, A., Saldfas, M. S., Bates, C. C., Molinaro, A., and Valvano, M. A. (2009). Biosynthesis and structure of the Burkholderia cenocepacia K56-2 lipopolysaccharide core oligosaccharide: Truncation of the core oligosaccharide leads to increased binding and sensitvity to polymyxin B. *Journal of Biological Chemistry* 284, 21738–21751. doi: 10.1074/jbc.M109.008532

Ruest, M. K., Supina, B. S. I., and Dennis, J. J. (2023). Bacteriophage steering of Burkholderia cenocepacia toward reduced virulence and increased antibiotic sensitivity. *J. Bacteriol.* 205. doi: 10.1128/jb.00196-23

Seed, K. D., and Dennis, J. J. (2005). Isolation and characterization of bacteriophages of the Burkholderia cepacia complex. *FEMS Microbiol. Lett.* 251, 273–280. doi: 10.1016/j.femsle.2005.08.011

Seed, K. D., and Dennis, J. J. (2008). Development of Galleria mellonella as an alternative infection model for the Burkholderia cepacia complex. *Infect. Immun.* 76, 1267–1275. doi: 10.1128/IAI.01249-07
